# Nisin lantibiotic prevents NAFLD liver steatosis and mitochondrial oxidative stress following periodontal disease by abrogating oral, gut and liver dysbiosis

**DOI:** 10.1038/s41522-024-00476-x

**Published:** 2024-01-17

**Authors:** Ryutaro Kuraji, Changchang Ye, Chuanjiang Zhao, Li Gao, April Martinez, Yukihiro Miyashita, Allan Radaic, Pachiyappan Kamarajan, Charles Le, Ling Zhan, Helene Range, Masataka Sunohara, Yukihiro Numabe, Yvonne L. Kapila

**Affiliations:** 1 Orofacial Sciences Department, School of Dentistry, University of California, San Francisco, San Francisco, CA USA; 2 Department of Periodontology, The Nippon Dental University School of Life Dentistry at Tokyo, Tokyo, Japan; 3 State Key Laboratory of Oral Diseases, National Clinical Research Center for Oral Diseases, Department of Periodontology, West China School of Stomatology, Sichuan University, Chengdu, China; 4 Department of Periodontology, Guanghua School of Stomatology, Hospital of Stomatology, Sun Yat-sen University, Guangzhou, China; 5 Sections of Biosystems and Function and Periodontics, School of Dentistry, University of California Los Angeles, Los Angeles, CA USA; 6 Department of Periodontology, University of Rennes, UFR of Odontology; Service d’Odontologie, CHU de Rennes, Rennes, France; 7 INSERM CHU Rennes, Institut NUMECAN (Nutrition Metabolisms and Cancer); CIC 1414, Rennes, France; 8 Department of Anatomy, The Nippon Dental University School of Life Dentistry at Tokyo, Tokyo, Japan

**Keywords:** Antimicrobials, Microbiota

## Abstract

Oral microbiome dysbiosis mediates chronic periodontal disease, gut microbial dysbiosis, and mucosal barrier disfunction that leads to steatohepatitis via the enterohepatic circulation. Improving this dysbiosis towards health may improve liver disease. Treatment with antibiotics and probiotics have been used to modulate the microbial, immunological, and clinical landscape of periodontal disease with some success. The aim of the present investigation was to evaluate the potential for nisin, an antimicrobial peptide produced by *Lactococcus lactis*, to counteract the periodontitis-associated gut dysbiosis and to modulate the glycolipid-metabolism and inflammation in the liver. Periodontal pathogens, namely *Porphyromonas gingivalis*, *Treponema denticola*, *Tannerella forsythia* and *Fusobacterium nucleatum*, were administrated topically onto the oral cavity to establish polymicrobial periodontal disease in mice. In the context of disease, nisin treatment significantly shifted the microbiome towards a new composition, commensurate with health while preventing the harmful inflammation in the small intestine concomitant with decreased villi structural integrity, and heightened hepatic exposure to bacteria and lipid and malondialdehyde accumulation in the liver. Validation with RNA Seq analyses, confirmed the significant infection-related alteration of several genes involved in mitochondrial dysregulation, oxidative phosphorylation, and metal/iron binding and their restitution following nisin treatment. In support of these in vivo findings indicating that periodontopathogens induce gastrointestinal and liver distant organ lesions, human autopsy specimens demonstrated a correlation between tooth loss and severity of liver disease. Nisin’s ability to shift the gut and liver microbiome towards a new state commensurate with health while mitigating enteritis, represents a novel approach to treating NAFLD-steatohepatitis-associated periodontal disease.

## Introduction

Periodontal disease, a common chronic inflammatory disease of the oral cavity, is caused by the host immune response to an oral polymicrobial dysbiosis present within oral biofilms^1,2^. These dysbiotic biofilms, which are predominantly comprised of anaerobic Gram-negative bacteria, namely periodontopathic bacteria, are continually releasing a lipopolysaccharide (LPS) challenge and other microbial molecules that trigger an altered host immune response and the release of tissue destructive enzymes in the periodontal tissues, thereby leading to periodontal tissue destruction and tooth loss^2,3^. These local microbial and inflammatory products from inflamed periodontal tissues travel into the systemic circulation^4–7^, and thereby are thought to be associated with systemic diseases via several different mechanisms^3^. In fact, periodontal disease has been known to exacerbate various metabolic disorders, such as obesity, diabetes, dyslipidemia, and cardiovascular disease^8–11^.

In recent years, growing evidence from basic^12–16^, clinical^17,18^, and epidemiological^18–23^ studies supports that periodontitis is associated with liver disease. In particular, non-alcoholic fatty liver disease (NAFLD), the hepatic manifestation of metabolic syndrome, has been closely associated with periodontal disease^24^. NAFLD is characterized by hepatic lipid deposition in the absence of a secondary predisposing factor, such as a habitual drinking history, viral infections or autoimmune diseases^25–28^. A portion of NAFLDs can develop into more severe and progressive forms, namely nonalcoholic steatohepatitis (NASH)^27^, further leading to cirrhosis and hepatocellular carcinoma, which are end-stage liver diseases^29,30^.

Enteral translocation of oral bacteria and inflammatory mediators, and gut microbial dysbiosis have been proposed as potential mechanisms that mediate this pathogenesis of NAFLD^31,32^. Based on the unique anatomical characteristics of the liver, all blood from the gut travels through the portal vein to gather into the liver before the blood reaches the systemic circulation^33,34^. Since gut dysbiosis increases the amount of hepatotoxins, such as LPS, ethanol, and volatile organic compounds^35–38^, and further enhances intestinal permeability by impairing intercellular tight junctions in the gut wall, it thereby promotes the translocation of hepatotoxins and enterobacteria and their byproducts to the liver^39,40^.

Indeed, several studies have reported that compared to individuals with periodontal health, the gut microbiome of patients with severe periodontitis is characterized by differences in microbial composition, low diversity, and a change in the ratio of Firmicutes/Bacteroidetes^41–43^. Similarly, in animal models, oral administration of periodontopathic bacteria caused alterations in gut microbiome composition as well as in glucose and lipid metabolism, leading to insulin resistance and hepatic lipid deposition^32,44–46^. *P. gingivalis*-induced gut dysbiosis further downregulated the expression of tight junction proteins, which play a role in gut barrier function, and increased serum LPS levels^32,47^. Therefore, potential liver damage derived from periodontitis-regulated gut dysbiosis may be mediated in the liver via the enterohepatic circulation and it may promote the progression of liver disease.

These facts support the premise that a realignment of the oral and gut dysbiosis towards a healthy state may prevent liver disease^24,48^. Therefore, a microbiome-targeted therapy using probiotics and bacteriocins have been proposed as novel strategies for manipulating the gut microbiome in the management of NAFLD^49–51^. Probiotics are defined as live cultured microorganisms that provide health benefits in humans and animals^52^, and bacteriocins are a generic term for antimicrobial peptides produced by the probiotic bacteria. A recent meta-analysis by Sharpton et al.^49^ revealed that the application of probiotics significantly improved liver-specific markers of hepatic function, liver stiffness, and steatosis in NAFLD patients. Preclinical animal studies have also shown that probiotics suppress the development of hepatic inflammation, insulin resistance, and fatty deposition by regulating the gut microbiota^53–56^.

However, little is known about the significance of probiotics and their bacteriocins in the management of liver pathology in patients with periodontal disease. Recently, our studies demonstrated that an antimicrobial peptide, nisin, which is produced primarily by *Lactococcus lactis* species, has effectiveness in the context of periodontal disease^57–60^. Nisin has received a lot of attention in the food industry and the medical field because of its potent and broad-spectrum activity even at trace concentrations, low host cell cytotoxicity at antibacterial concentrations, and low likelihood of promoting the development of bacterial resistance^60–64^. Nisin, also classified as a Class I bacteriocin, is known as a lanthionine-based (lanthionine-containing peptides) antimicrobial based on its chemical structure, because it has unique amino acids that are caused by translational modifications^62,65,66^. Furthermore, our data revealed that nisin and a nisin-producing probiotic *Lactococcus lactis* decreased the number of pathogenic bacteria while retaining oral commensal bacteria, such as *Neisseria* species within salivary-derived biofilms in vitro^59^. In this context, nisin also significantly inhibited the formation, structure, and viability of biofilms spiked with periodontopathic bacteria and shifted the microbiome composition back toward the healthy control state. We further found that in a polymicrobial infection mouse model of periodontal disease, oral administration of the probiotic *L. lactis* or its bacteriocin nisin also promoted a shift toward a healthy oral microbiome while preventing gingival inflammation and alveolar bone loss^57,58^. In the present study, the same polymicrobial mouse model^67^ was induced by oral infection with *P. gingivalis*, *Fusobacterium nucleatum, Treponema denticola*, and *Tannerella forsythia*, and employed to evaluate the effects of nisin in modulating the dysbiosis in the oral, gut and liver microbiome and associated gastrointestinal and liver pathophysiology.

## Results

### Nisin shifts a disease-associated microbiome toward a new composition commensurate with health in various organs

**Fig. 1:**
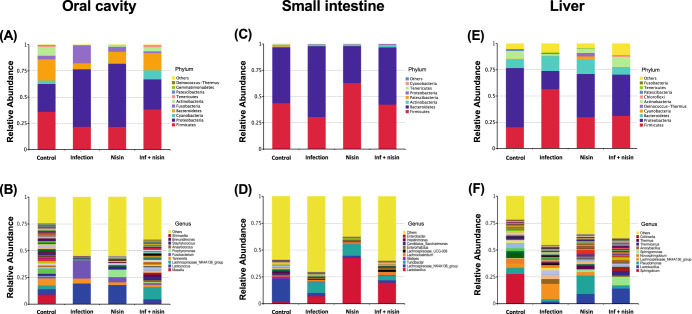
Nisin promotes a shift from a disease-associated microbiome toward a healthy state through multiple body sites. Bar graphs show relative abundance of each bacteria taxa at the phylum level (**A**, **C**, **E**) and genus level (**B**, **D**, **F**) in oral cavity, small intestine, and liver (*n* = 6). The list next to the bars shows the top 10 taxa relative abundance in each taxonomic rank.

To determine the extent to which nisin modifies the microbiome of the oral cavity, small intestine, and liver following an oral polymicrobial infection, the microbial composition and abundance at these sites were analyzed by 16 S rRNA sequencing at three taxonomic levels, namely at the phylum, genus, and species level (*n* = 6). Figure 1 shows the relative abundance in taxa rank at the phylum and genus level. In particular, focusing on the genus level, the data included 162 genera in the oral cavity, 53 in the intestinal feces, and 154 in the liver (Supplementary Table 1), while the “others” group, indicated by the yellow bar, were unidentified or unclassified bacterial genera. Less than 10% of these did not match any known phylum at the phylum level. In other words, more than 90% of all genera observed belonged to a known phylum.

**Fig. 2:**
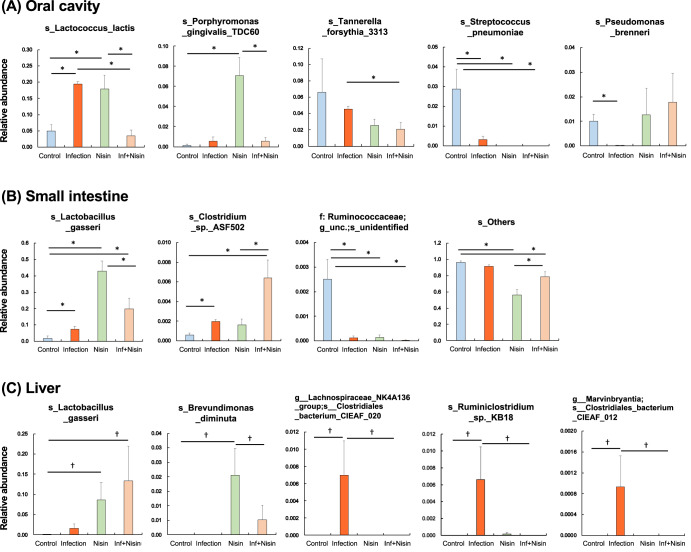
Nisin shifted the microbiome from a disease-associated state toward a healthy state through multiple body sites. Bar plots show bacterial taxa that exhibited significant differences in relative abundances at the species level within the oral cavity (**A**), small intestine (**B**) and liver (**C**). The data in the bar graphs are shown as means ± standard deviation. *p < 0.05 between groups with Tukey test, †*p* < 0.05 with *t* test (*n* = 6).

In the oral cavity, (Fig. 1A and Supplementary Fig. 1A), the relative abundance of the phylum Proteobacteria and Fusobacteria were significantly increased in the infected mice compared to the healthy control mice, but the proportions of Bacteroidetes, Firmicutes, Cyanobacteria, and Actinobacteria were decreased. In contrast, nisin treatment significantly prevented these oral microbiome changes and shifted the microbial composition toward a healthy new state that was different from controls. At the genus level (Fig. 1B and Supplementary Fig. 2A), the proportions of *Lactococcus* and *Fusobacterium* significantly increased in the infection group compared to the control group, whereas the abundance of *Lachnospiraceae NK4A136*, *Streptococcus*, *Cutibacterium*, *Granulicatella*, and *Veillonella* decreased. However, nisin recovered the disease-associated changes in *Lactococcus*, *Fusobacterium*, and *Lachnospiraceae NK4A136* back toward the control healthy state. The administration of nisin alone increased *Lactococcus* and *Porphyromonas*, and reduced the proportions of *Lachnospiraceae NK4A136*, *Streptococcus*, *Granulicatella*, and *Veillonella*. At the species level (Fig. 2A), there was an increase in *Lactococcus lactis* and a decrease in *Streptococcus pneumoniae* and *Pseudomonas brenneri* in the infection group but a preventive effect was noted in the infection+nisin group, which was consistent with the microbial findings observed at the genus level.

In the gut, analysis of the relative abundance of microbiota in small bowel feces at the phylum level (Fig. 1B and Supplementary Fig. 1B) revealed a lower amount of Firmicutes and higher amount of Bacteroidetes in the infection group than in both the control and infection+nisin groups. In contrast, Actinobacteria exhibited a higher abundance in all other three groups compared to the control group, and Tenericutes was lower. At the genus level (Fig. 1D and Supplementary Fig. 2B), 15 bacterial taxa showed significant changes among groups. In particular, nisin significantly increased the acetate- and butyrate-producing beneficial bacterium, such as *Lactobacillus*, *Lachnospiraceae UCG-001 group*, *Lachnospiraceae UCG-006 group*, *Lachnoclostridium* and *Acetitomaculum*. However, the infection group showed a significant increase in the proportion of *Turicibacter* and *Bifidobacterium*, which was prevented by nisin treatment. Interestingly, these genera taxa, which showed significant changes, are predominantly classified in the Firmicutes phylum. Moreover, at the species level (Fig. 2B), the proportion of *Lactobacillus gasseri* was markedly increased in both the nisin and infection+nisin groups. *Clostridium sp. ASF502* and an unidentified bacteria belonging to unclassified-*Ruminococcaceae* also showed significant alterations among groups.

In the liver tissue, microbiome changes at the phylum level revealed a significantly higher abundance of Firmicutes in the infection group than in the control group, whereas Proteobacteria and Actinobacteria were lower (Fig. 1E and Supplementary Fig. 1C). Bacteroides showed no significant difference among groups. Importantly, nisin treatment consistently maintained the same relative abundance phylum levels as the healthy controls, thus protecting this organ from the disease-related changes in the microbiota. In addition, at the genus level (Fig. 1F and Supplementary Fig. 2C), the proportion of *Lachnospiraceae NK4A136 group* and *Turicibacter*, which are classified in the phylum Firmicutes, tended to be higher in the infection group compared to the control and infection+nisin groups. In contrast, in terms of the phylum Proteobacteria, the abundance of genus *Sphingobium* dramatically decreased following the infection and/or nisin treatment, and *Psuedomonas*, *Brevundimonas*, and *Massilia* were significantly increased by nisin treatment alone. As shown in Fig. 2C for the liver tissue, at the species level, the proportion of *Lactobacillus gasseri* was markedly higher in both the nisin and infection+nisin groups, revealing a similar tendency as in the gut microbiome. In contrast, a higher abundance of *Clostridiales bacterium CIEAF 020*, *Ruminoclostridium sp. KB18*, and *Clostridiales bacterium CIEAF 012* were detected in the infection group compared to the other groups (Fig. 2C); although these bacterial taxa showed significant differences at *P* < 0.05 (unpaired *t* test), these differences did not reach further significance when adjusted by False Discovery Rate (FDR).

### Nisin prevents the oral polymicrobial infection-mediated alterations in microbial diversity and community structure in the oral cavity and liver

**Fig. 3:**
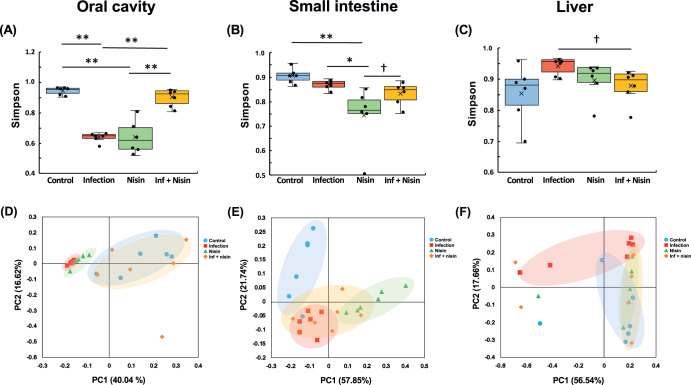
Nisin prevents the alterations in microbial diversity and community structure induced by a polymicrobial infection in the oral cavity, small intestine, and liver. Simpson index for analysis of alpha diversity (**A**–**C**). PCoA using weighted UniFrac distance for evaluating differences in microbiome composition between groups (**D**–**F**). Box-plots display the median, first quartiles (25th percentile), third quartiles (75th percentile), and minimum and maximum whiskers, with all data points plotted. *p < 0.05 and **p < 0.01 between groups with Tukey test, ^†^*p* < 0.05 with *t* test (*n* = 6).

As shown in Supplementary Fig. 3, rarefaction curves based on number of observed species as alpha diversity metrics indicated that 6000–8000 sequences per sample are sufficient for capturing the characteristics of microbial communities in both oral cavity, small intestine, and liver (*n* = 6). Thus, in order to assess the changes in bacterial diversity following the polymicrobial infection and nisin treatment, the Simpson diversity index was analyzed based on the OTU numbers from the oral cavity, small intestine and liver, respectively. As shown in Fig. 3A, the bacterial diversity scores for the oral microbiome were significantly lower in both the infection and nisin groups compared to the control group (*P* < 0.001; *n* = 6; Tukey test). The infection+nisin group had a higher diversity score compared to the infection and nisin groups (*P* < 0.001), but equivalent to the control group (*P* = 0.115), showing nisin’s ability to recover the microbiome diversity back toward a state of health like the control. In the small intestine (Fig. 3B), the diversity was significantly lower in the nisin group (*P* < 0.003 and *P* < 0.023, respectively) compared to the control and infection groups. However, the diversity of the infection+nisin group was slightly increased (*P* < 0.148) compared to the nisin group, although this was not significant and intermediate between the infection and nisin groups. In contrast to the oral and gut microbiome, the bacterial diversity in the liver tissue (Fig. 3C) tended to increase following the polymicrobial infection, but this change was mitigated by nisin treatment. Although there was no statistical difference in these microbiome changes among groups when using the Tukey test, the *t* test showed a significant difference between the infection and inf+nisin group (*P* < 0.038).

To evaluate the overall similarity and dissimilarly in the microbiome changes among different treatment groups, we further performed Coordinate Analysis (PCoA) using weighted UniFrac distance. For the oral cavity (Fig. 3D), the microbiome composition of the infection and nisin groups revealed significant qualitative differences compared to the control group (*P* = 0.002 with *R* = 0.856 and *P* = 0.002 with *R* = 0.907, respectively; *n* = 6; analysis of similarity). However, among infected animals, those treated with nisin were more similar to the control, and there was a significant difference between the infection and the inf + nisin groups (*P* < 0.01 with *R* = 0.191). On the other hand, in the small intestine (Fig. 3E), the microbial composition of all treatment groups (the infection: *P* = 0.011 with *R* = 0.528, the nisin: *P* = 0.003 with *R* = 0.802, and the inf + nisin: *P* = 0.003 with *R* = 0.557, respectively) were significantly different from the control group. Also, the gut microbiome of the nisin group exhibited a shift to a different state from that of the infection group (*P* = 0.031 with *R* = 0.279). Interestingly, nisin treatment of infected mice further induced a change in the gut microbiome toward a middle state between the infection and nisin groups, which was consistent with the previously mentioned findings for the Simpson diversity. Similarly, for the liver (Fig. 3F), the microbiome composition of the infection group was significantly different from the control and nisin groups (*R* = 0.527, *P* = 0.011 and 0.279, *P* = 0.031, respectively). We again found that among the infected mice, those treated with nisin were similar to the control group, indicating that nisin also drives the overall hepatic microbiome composition toward the healthy control state as in the oral and GI tissues.

### Periodontal inflammation following polymicrobial oral infection is reduced by nisin

**Fig. 4:**
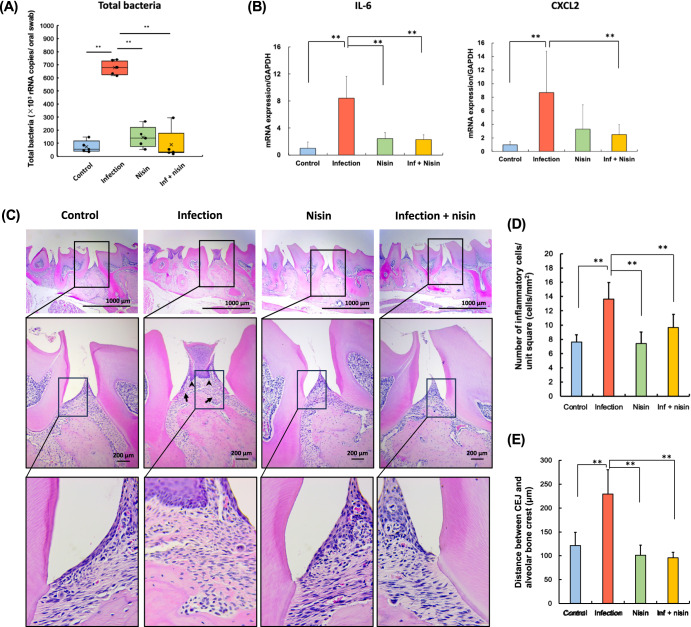
Periodontal inflammation following polymicrobial oral infection is reduced by nisin. **A** Total bacteria amount, (**B**) Gene expression of pro-inflammatory cytokine, and (**C**) Histopathological evaluation in periodontal tissue of the maxillary specimens (Scale bar: 1000 μm in the upper panel and 200 μm in the lower panel, respectively). **D** The number of inflammatory cells per 1.0 mm^2^ of connective tissue, and (**E**) Average calculated linear distance between the CEJ and ABC in histological image. The data in the bar graphs are shown as means ± standard deviation. Box-plots display the median, first quartiles (25th percentile), third quartiles (75th percentile), and minimum and maximum whiskers, with all data points plotted. *p < 0.05 and **p < 0.01 between groups with Tukey tests for real-time PCR analysis (*n* = 5) and histological analysis (*n* = 3), respectively.

To evaluate nisin’s ability to alter the host inflammatory response that mediates periodontal disease, we analyzed the immune cytokine profiles within gingival tissues via changes in gene expression, histological inflammatory cell infiltrate in the periodontal tissues, and alveolar bone height, while validating changes in the total bacterial load in the oral cavity (Fig. 4, Supplementary Fig. 4, 5). The number of total oral bacteria were significantly higher in the infection group compared to the control group as assessed by real-time polymerase chain reaction (RT-PCR) of oral swabs (Fig.4A) (*P* < 0.001; *n* = 5; Tukey test). However, among the infected animals, nisin markedly decreased the total bacterial count similar to the control levels (*P* < 0.001). For the gene expression analyses (Fig. 4B), the levels of interleukin (IL)-6 and C-X-C Motif Chemokine Ligand 2 (CXCL2) were significantly upregulated in the infection group (*P* < 0.001 and *P* < 0.01, respectively; *n* = 5; Tukey test) compared to the control. However, nisin treatment significantly prevented these cytokine changes in the infection group (*P* < 0.001 and *P* < 0.05, respectively). Other cytokine-related genes, including tumor necrosis factor (TNF)-α, C-C Motif Chemokine Ligand 2 (CCL2), IL-1β, transforming growth factor (TGF)-β, and interferon (IFN)-γ, showed a similar tendency, but these differences did not reach statistical significance (Supplementary Fig. 5). Next, we evaluated the effects of the infection and nisin treatment on the morphologic features and inflammatory cell infiltrate of the periodontal tissues using hematoxylin and eosin (HE)-stained histological sections (Fig. 4C). In the control group, dense collagen fiber bundles were organized orderly in a certain direction and very few inflammatory cells were localized in the gingival connective tissue just below the thin junctional epithelium. In contrast, the gingival tissues from the infection group exhibited epithelial hyperplasia with rete ridge elongation and an infiltration of numerous inflammatory cells into the lamina propria (*p* < 0.001; *n* = 3; Tukey test; Fig. 4D). Compared to the control group, a disordered orientation of the connective tissue fibers and marked dilatation of capillary blood vessel were also observed in the infection group. In addition, several osteoclasts were present on the rough alveolar bone surface in the infection group. Importantly, treatment with nisin improved the rete ridge elongation in the epithelial tissue, fiber orientation and vascular dilation, and significantly reduced the inflammatory cell infiltrate (*p* < 0.001), which were observed in the infection group, as previously reported by our group^57^.

The linear distance between the cemento-enamel junction (CEJ) and the alveolar bone crest (ABC) was 121.18 ± 27.77 μm in the control group. Compared with the control group, the CEJ-ABC distance was significantly increased in the infection group (229.28 ± 51.28 μm) (*p* < 0.05, *n* = 3; Tukey test; Fig. 4E). In contrast, the distance in the nisin group (101.33 ± 21.18 μm) and inf + nisin group (95.52 ± 12.10 μm) was not significantly different from that in the control group, although the distance was significantly less when compared to the infection group.

### Inflammation in small intestine following polymicrobial oral infection is prevented with nisin treatment

**Fig. 5:**
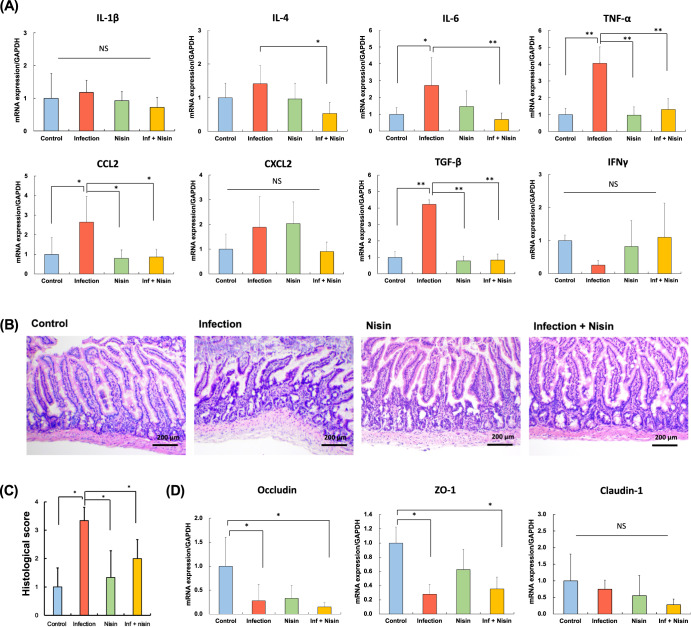
Inflammation of small intestine following polymicrobial oral infection is prevented with nisin treatment. **A** Gene expression of immune cytokine profiles from the ileum tissue, (**B**) Histopathological examination to evaluate severity of inflammation of small intestine (Scale bar: 200 μm), (**C**) Histological score, (**D**) Gene expression of tight junction proteins associated with gut barrier function. The data in the bar graphs are shown as means ± standard deviation. *p < 0.05 and **p < 0.01 between groups with Tukey test for real-time PCR analysis (*n* = 5) and Steel-Dwass test for histological analysis (*n* = 3), respectively.

To evaluate the effect of the oral polymicrobial infection and nisin treatment on the small intestine, we performed gene expression assays to evaluate the immune cytokine levels in the ileum (Fig. 5). The gene expression levels of the pro-inflammatory cytokines, including IL-6, TNF-α, and CCL2, were significantly elevated in the infection group compared to the control group (Fig. 5A**;** *P* < 0.01 and *P* < 0.05, respectively; *n* = 5; Tukey test). However, this cytokine upregulation in the infection group was suppressed by nisin treatment. The gene expression levels of the anti-inflammatory cytokines, IL-4 and TGF-β, showed similar significant changes like the pro-inflammatory cytokines, which may reflect a resolution of inflammation and pro-resolving response.

The severity of the inflammatory changes in the small intestine were further evaluated by histopathological examination. In the infected animals, compared to the control group, the ileum showed marked wall thickening with an exfoliation of the mucosal epithelium, decreased villi height, and a severe inflammatory cellular infiltrate into the lamina propria (Fig. 5B). Conversely, the intestinal tissues from the nisin-treated infected animals had a mild inflammatory cell infiltration of the mucosa. Furthermore, nisin treatment significantly improved the villi height and histological scores in terms of the inflammatory cell infiltrate, vascular density, loss of goblet cells, and thickening of the intestinal wall (*p* < 0.05; *n* = 3; Steel-Dwass test; Fig. 5C).

Moreover, we used RT-PCR to evaluate the gene expression of tight junction proteins, which are responsible for regulating intestinal barrier function and intestinal permeability (Fig. 5D).

The expressions of *Occludin* (*Ocln)* and *Tight junction protein-1* (*Tjp1)* were significantly downregulated in the small intestine following the polymicrobial oral infection (*P* < 0.05; *n* = 5; Tukey test). However, among the infected mice, this downregulation was also present in the infection+nisin group, and was not prevented by treatment with nisin, although some positive trends were noted for *Tjp1*/ZO-1. For *Claudin-1* (*Cldn1)* gene, there were no significant changes among groups.

### Nisin treatment attenuates the total bacterial burden and periodontal pathogens that enter the small intestine and liver

**Fig. 6:**
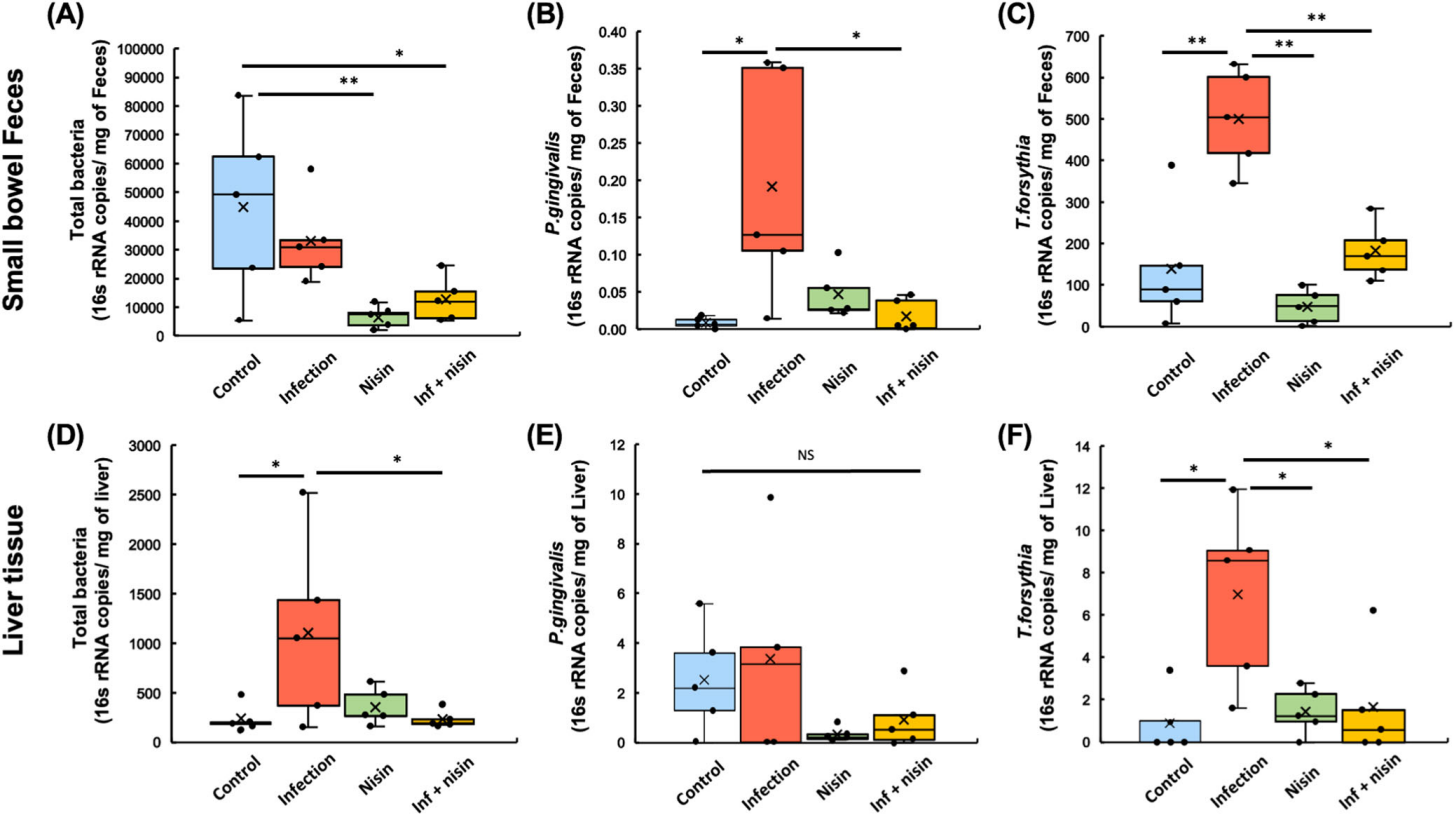
Nisin treatment attenuates the burden of total bacterial and periodontal pathogens into the small intestine and liver. In order to further assess the bacterial load on gut-liver-axis, the number of total bacteria and periodontal pathogens was measured in the small bowel feces (**A**–**C**) and liver samples (**D**–**F**) using RT-PCR in the manner of absolute quantification. Box-plots display the median, first quartiles (25th percentile), third quartiles (75th percentile), and minimum and maximum whiskers, with all data points plotted. *p < 0.05 and **p < 0.01 between groups with Tukey test (*n* = 5).

In order to further assess the bacterial load on the oral-gut-liver-axis, the number of total bacteria and periodontal pathogens were measured in the small bowel feces and liver samples using RT-PCR to determine their absolute quantification (Fig. 6). For the small bowel feces, the copy number of the universal 16S rRNA gene (total bacteria) was significantly reduced following nisin treatment both in the presence and absence of the polymicrobial infection (*P* < 0.05; *n* = 5; Tukey test; Fig. 6A). Interestingly, in the small bowel feces, *P. gingivalis* and *T. forsythia* exhibited significantly higher levels in the infection group than the control group (*P* < 0.05 and *P* < 0.001, respectively; Fig. 6B, C), whereas nisin markedly decreased the levels of these periodontal pathogens among the infected animals (*P* < 0.05 and *P* < 0.001, respectively). Moreover, in the liver tissue, the number of total bacteria was significantly elevated in the infection group compared to the control group, and this increase was prevented by nisin treatment in the infected animals (*P* < 0.05; Fig. 6D). Similar to the small bowel feces, *T. forsythia* in the liver of the infected group (*P* < 0.05) was significantly higher than in all the other groups, and nisin abrogated its increase in the infected mice (Fig. 6F). *P. gingivalis* also tended to decrease in the infected group when treated with nisin, although this decrease did not reach statistical significance (Fig. 6E). However, the other oral periodontal pathogens that were part of the oral polymicrobial infection, *F. nucleatum* and *T. denticola*, were not detected in the feces or liver of the infected mice (Data not shown).

### Hepatic lipid deposition by polymicrobial infection is significantly abrogated in mice treated with nisin

**Fig. 7:**
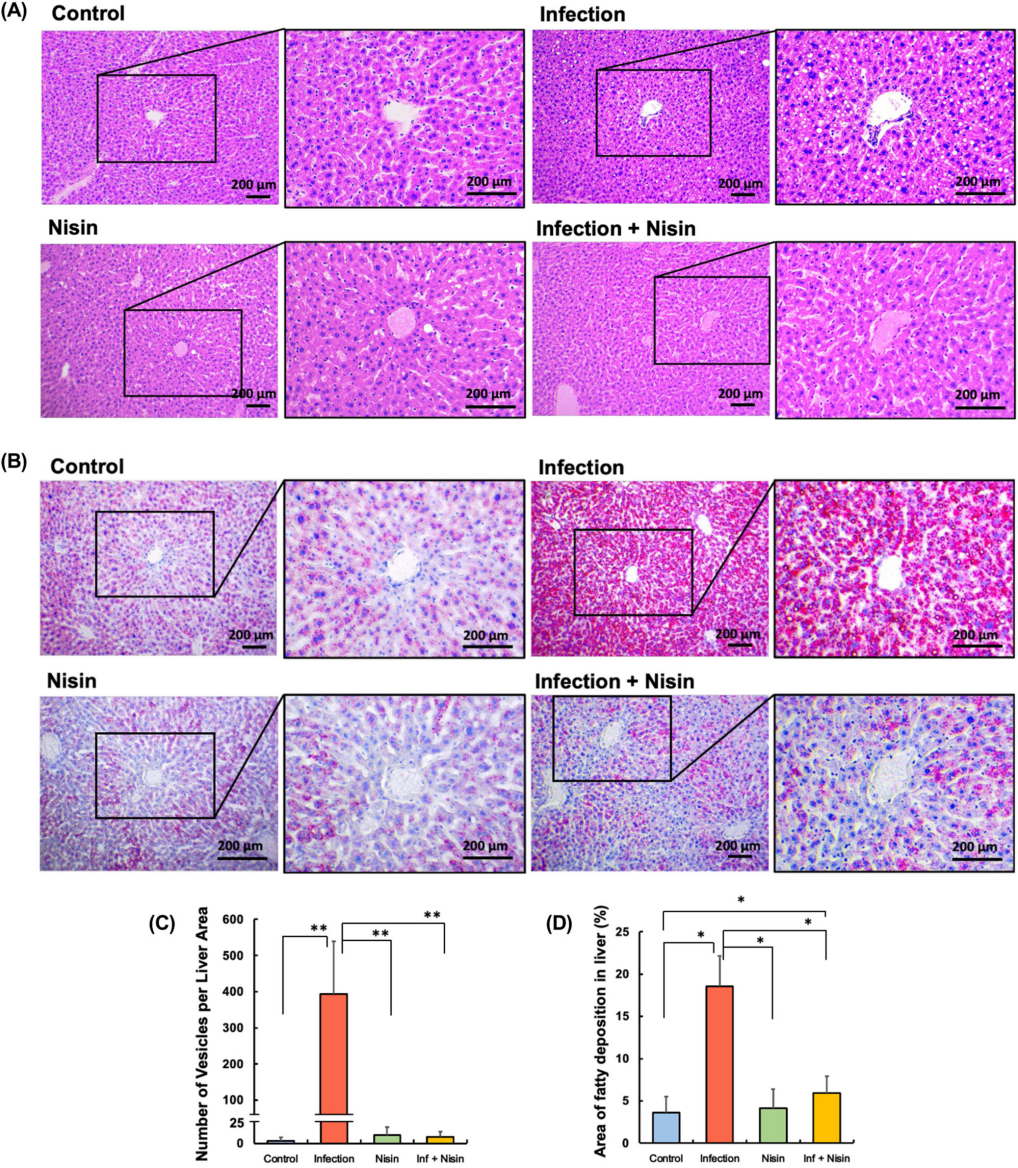
Hepatic lipid deposition by polymicrobial infection is significantly abrogated in mice treated with nisin. Histopathological analysis of liver tissue was conducted to evaluate the ability of nisin to modulate the diseased changes in lipid deposition and inflammatory reaction in the histological images stained by hematoxylin (**A**) and Oil red (**B**) (Scale bar: 200 μm). Four different fields (×100 magnification) were randomly selected on the images of three tissue sections per mouse specimen (*n* = 3 per group), and number of vesicles (**C**) and area of orange-stained fatty deposition (**D**) was measured using ImageJ analysis software. The data in the bar graphs are shown as means ± standard deviation. *p < 0.05 between groups and **p < 0.01 between groups with Dunn’s test or Tukey test.

Histopathological analysis of the liver tissue was conducted to further evaluate the effects of the oral polymicrobial infection and nisin treatment on the liver. As shown in the histological images of the representative liver tissues stained with HE (Fig. 7A) and Oil Red O (Fig. 7B), limited lipid deposition and infiltrating inflammatory cells were observed in the healthy control animals. In contrast, the infection group showed an obvious and severe lipid deposition, a number of perivenous small lipid droplets with vesicles, and detachment of endothelial cells in the central vein. However, in the inf + nisin group, only limited lipid deposition and vesicles were observed similar to the control and nisin groups, indicating that nisin improved the lipid metabolic changes in the liver following the polymicrobial infection.

Hematoxylin stained liver sections were analyzed using image analysis software in order to quantify the number of vesicles. In agreement with the other histological findings, the polymicrobial infection significantly increased the number of vesicles by 103-fold (from 3.8±4.7 vesicles in the control group to 393.4 ± 146.1 vesicles in the infection group; *P* < 0.0001; *n* = 3; Dunn’s test; Fig. 7C). However, nisin significantly decreased the number of vesicles in the infection group (*P* < 0.01), with no significant difference compared to the control group (*p* = 0.5674; 3.8±4.7 vesicles in the control group versus 11.30 ± 11.36 in the infection + nisin group). To further quantify the degree of lipid deposition, oil red O-stained liver sections were analyzed. Similar to the findings for the increased vesicle numbers, the area of hepatic lipid deposition significantly increased by 15% in the infection group (*P* < 0.05; *n* = 3; Tukey test; Fig. 7D). Whereas, among the infected animals, nisin markedly decreased the lipid deposition (*P* < 0.05), although there was still a significant difference between the infection+nisin group and the control group (*P* < 0.05).

### Nisin restores changes in gene expression related to hepatic mitochondrial function and oxidative stress following polymicrobial infection

**Fig. 8:**
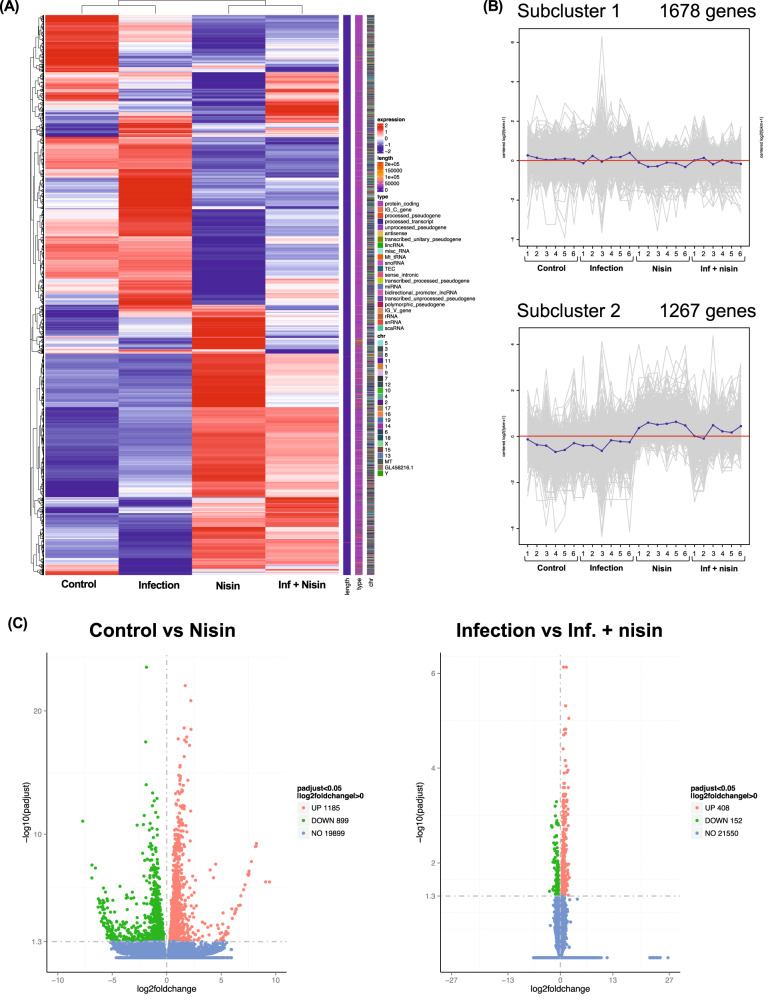
Gene profile in liver tissue analyzed by RNA sequencing. **A** RNA-seq detected 2,949 genes were categorized by hierarchical clustering approach (*n* = 6). **B** Subcluster analysis were further performed to determine the trends in gene expressions among groups. **C** Volcano plot revealed 2,084 DEGs (1185 up-regulated and 899 down-regulated) between the control group and the nisin group (left panel) and 560 DEGs (408 up-regulated and 152 down-regulated) between the infection group and the inf. + nisin group (right panel).

RNA sequencing of mouse liver tissue was performed to explore the underlying mechanisms of the regulatory action of Nisin in fatty liver disease induced by polymicrobial infection. A total of 18,614,688-30,662,424 (mean 22,562,974) paired-end clean reads per sample (*n* = 6) were generated by the Illumina MiSeq system. The distribution of fragments per kilobase of exon per million mapped fragments (FPKM) for all genes (54,532 genes) was consistent across all samples, but the majority of genes had low expression (FPKM < 1.0). After removing the low-expressed genes between groups, a final set of 2,949 genes was used in this study (Fig. 8A). Among these genes, two gene subclusters were detected that showed different expression patterns (Fig. 8B). The expression level of 1678 genes in subcluster 1 tended to be upregulated in the infection group and downregulated in the nisin and the inf + nisin groups compared to the control group. On the other hand, the expression of 1267 genes in subcluster 2 tended to be increased in the nisin and the inf + nisin groups compared to the control and the infection groups. Therefore, to detect genes that may be important regulators of the effect of nisin in the liver, we performed a differentially expressed genes (DEGs) analysis (fold change ≥ 1.3, *q*-value < 0.05; *n* = 6; pairwise *t* test) between the control and the nisin group or between the infection and the inf + nisin group, resulting in 2,084 and 560 genes, respectively (Fig. 8C).

**Fig. 9:**
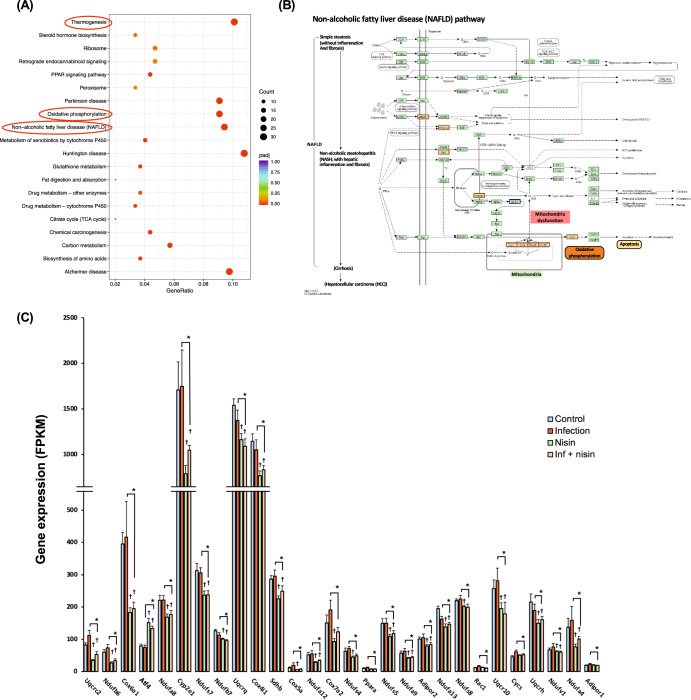
Functional analysis expressed genes in liver tissue for KEGG pathways. **A** Scatter Plot shows for top 20 significantly enriched KEGG terms, which were determined between the infection group and the inf. + nisin group by ClusterProfiler (version 3.8.1). **B** The enriched pathway of Non-alcoholic fatty liver disease (NAFLD, term 04932) in KEGG database. **C** The bar graph revealed differential genes among four groups for the NAFLD-related pathway (* adjusted *p*-value by FDR < 0.05 with pairwise *t* test, *n* = 6). The data are shown as means ± standard deviation.

To examine the global function of genes whose expression was altered by nisin treatment in the polymicrobial infected mice, a gene functional analysis for Kyoto encyclopedia of genes and genomes (KEGG) pathways was performed. The top 20 significantly enriched KEGG terms (*q*-value < 0.05) were identified for the DEGs between the infection group and the inf + nisin group. KEGG terms related to energy metabolism, such as “thermogenesis”, “oxidative phosphorylation”, “PPAR signaling pathway”, and “metabolism of xenobiotics of cytochrome P450” were detected (Fig. 9A). Interestingly, the “non-alcoholic fatty liver disease (NAFLD)” term was detected with the fourth highest count and contained 28 genes classified as energy metabolism-related terms (Fig. 9B). Among these, genes related to electron transfer complexes in mitochondria (CxI: Ndufb6, Ndufa8, Ndufs7, Ndufb7, Ndufa12, Ndufs4, Ndufs5, Ndufa9, Ndufa13, Ndufs8, Ndufv1, Ndufa4; CxII: Sdhb; CxIII: Uqcrc2, Uqcrq, Uqcr10, Uqcrh; CxIV: Cox6a1, Cox4i1, Cox5a, Cox7a2) were more common (Fig. 9C). In addition, for other cell organelles, peroxisome-related genes (Adipor1, Adipor2, Ppara), Cytochrome P450-related genes (Cyp2e1) and Cytochrome c-related genes (Cycs) were significantly lower in the inf + nisin group than the infection group.

**Fig. 10:**
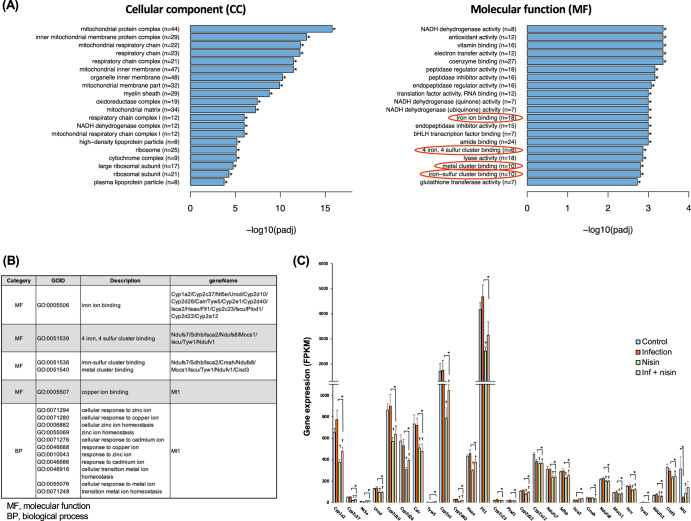
Functional analysis expressed genes in liver tissue for GO pathways. **A** Bar plot shows for top 20 significantly enriched GO terms at molecular function category, which were determined between the infection group and the inf. + nisin group by ClusterProfiler (version 3.8.1). **B** Related genes of iron binding and metal cluster binding in GO database. **C** The bar graph reveals differential genes among four groups for the iron binding-, metal cluster binding-, and cellular response to metal ion- related genes (*: adjusted i-value by FDR < 0.05 with pairwise *t* test, *n* = 6). The data are shown as means ± standard deviation.

Next, gene ontology (GO) enrichment analysis was performed between the infection and the inf + nisin groups to assess cellular structure and molecular function in more detail, and the top 20 significantly enriched GO terms (*q* value < 0.05) were identified (Fig. 10A). For GO terms at the cellular component level, genes related to mitochondrial protein complexes, membrane structure, and respiratory chain were more abundant in the infection group, consistent with KEGG pathway analysis. Furthermore, at the molecular function (MF) level, we detected GO terms related to the binding capacity of metal ions, such as iron and copper, in addition to NADH dehydrogenase, electron transfer and antioxidant activities. These metal ions play an important role in oxidative phosphorylation via electron transfer complexes in mitochondria, and the list in Fig. 10B shows the role of genes related to metal ion binding that have significant differences between groups in the GO database. Indeed, the expression of many genes in the iron and copper ion binding and iron-sulfur cluster binding term was enhanced in the infection group, while it was significantly attenuated in the inf + nisin group (Fig. 10C). In addition, the MT1 gene, which is involved in cellular response and homeostasis of metal ions, such as copper and zinc, was significantly downregulated in the infection group compared to the control group (Supplementary Fig. 6).

### Lipid peroxide deposition in the liver enhanced by polymicrobial infection is significantly inhibited in nisin-treated mice

**Fig. 11:**
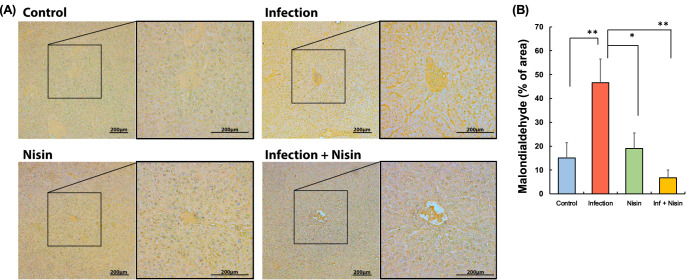
Hepatic malondialdehyde (MDA) deposition following the polymicrobial infection is significantly reduced in mice treated with nisin. MDA in liver tissue was quantified to evaluate the ability of nisin to modulate lipid peroxidation due to oxidative stress in the histological sections stained by immunohistochemistry (**A**) (Scale bar: 200 μm). Four different fields (100× magnification) were randomly selected on the images of three tissue sections per mouse specimen (*n* = 3 per group), and area of brown-stained MDA deposition (**B**) was measured using ImageJ analysis software. The data in the bar graphs are shown as means ± standard deviation. *p < 0.05 between groups and **p < 0.01 between groups with Dunn’s test.

Given the increased numbers of lipid vesicles, lipid deposition, and gene clusters related to NAFLD in the infection group, malondialdehyde (MDA) expression in liver tissue was further evaluated as an indicator of lipid peroxide and oxidative stress, especially because of the importance of genes related to mitochondrial oxidative phosphorylation. Brown-staining MDA was rarely observed in the inf + nisin group as well as the control and nisin groups (Fig. 11A). In the infection group, on the other hand, MDA deposition was more abundant within the cytoplasm, vesicles, and vascular sinusoids. Using semi-quantitative analysis, it was determined that the MDA deposition significantly increased by 3-fold in the polymicrobial infection group compared to control (from 15.08±6.37% in control to 46.65±9.90%; *p* < 0.01; *n* = 3; Dunn’s test; Fig. 11B). Reiterating previous results, MDA was significantly reduced in the inf + nisin group (6.76±3.30%, *p* < 0.0001).

### Human autopsy study

**Fig. 12:**
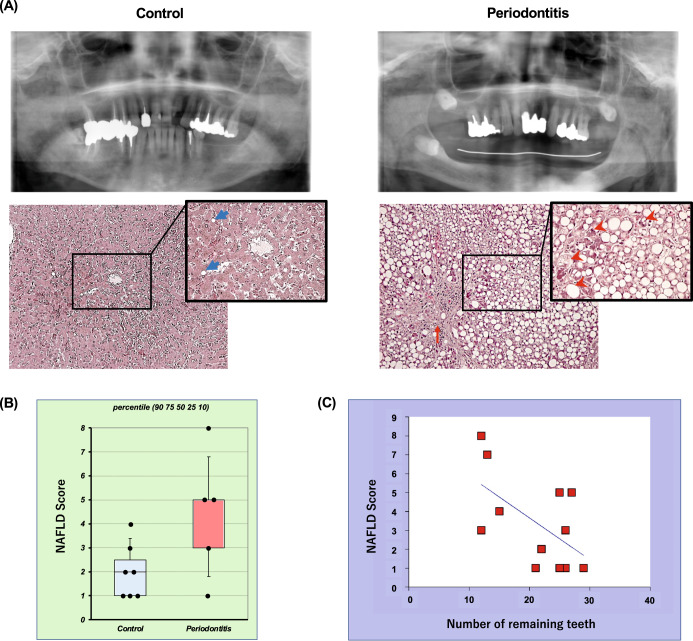
The number of remaining teeth correlated with the severity of liver disease at human autopsy study. The control and periodontitis groups were defined based on the severity and extent of periodontal disease assessed on panoramic radiographs and cone-beam CT images, respectively. Panel (**A**) shows representative findings of oral (upper panel) and liver (lower panel) in each group. Blue arrows indicate small fat droplets, red arrows indicate scarring fibrosis, and arrowheads indicate ballooning hepatocytes with cell injury, respectively. The NAFLD activity score (NAS) measured from histological findings (**B**) was compared between the control group (*n* = 7) and the periodontitis group using an unpaired *t* test (*n* = 5). Box-plots display the 90, 75th, 50th 25th, 10 percentile, respectively, with all data points plotted. The correlation coefficient between the NAS and the number of remaining teeth (**C**) was analyzed using the Pearson correlation coefficient (*n* = 12).

Additionally, a human autopsy observational study was conducted to provide stronger support for the relationship between periodontal disease and liver disease. In this study, a total of 19 Japanese humans, which included ten male cadavers (82–94 years old; mean, 88.1 years) and nine female cadavers (78–99 years old; mean, 87.1 years old) were initially examined. In regards to this information, due to issues with cause of death and number of remaining teeth, seven subjects were initially excluded from the study. Finally, the control (*n* = 7) and periodontitis (*n* = 5) groups were included for the analysis (Supplementary Table 2) and defined based on the severity of periodontal disease as assessed by the amount of alveolar bone resorption and the number of remaining teeth, respectively. Figure 12A shows representative findings for each group. The NAFLD score of the periodontitis group tended to be higher than that of the control group, but the difference was not significant (*p* = 0.054, unpaired *t* test, Fig. 12B). On the other hand, the number of remaining teeth showed a significant negative correlation with the NAFLD activity score (NAS) (*n* = 12, *r* = −0.58, *p* < 0.05, Pearson correlation coefficient, Fig. 12C).

With regard to bacterial detection, *F. nucleatum* was detected in 58.3% (7/12 cadavers) of the gingival tissues, *T. forsythia* in 33.3% (4/12 cadavers) and *T. denticola* in 8.3% (1/12 cadavers), but these detection rates were not significantly different between the control and the periodontitis groups (2 × 2 chi-square, *p* > 0.05). In liver tissue, only *F. nucleatum* was detected in 33.3% (4/12 cadavers) of samples, with no significant difference between groups (*p* > 0.05). Similarly, total bacterial counts in gingival tissue (the control group: 14.3 ± 3.3 copies / mg of tissue vs the periodontitis group: 25.8 ± 25.7 copies/mg of tissue) and liver tissue (the control group: 8.2 ± 3.2 copies/mg of tissue vs the periodontitis group: 11.6 ± 3.0 copies/mg of tissue) were measured by unpaired *t* tests, but showed no significant differences (*p* > 0.05).

## Discussion

A number of studies have recently highlighted that periodontal disease negatively affects glycolipid metabolism and immune responses in the liver via oral and gut dysbiosis^24,48^. In this regard, microbiome-targeted therapy using probiotics and bacteriocins may be an effective approach for shifting not only the periodontal disease-related oral dysbiosis but also the gut dysbiosis toward a healthy state and subsequently preventing the development and progression of liver disease, such as NAFLD and NASH. Therefore, in this study, nisin, a bacteriocin produced by *L. lactis*, was orally administered in the context of a polymicrobial periodontal disease mouse model, and the effects of nisin on gut dysbiosis and liver disease were evaluated, revealing the significant therapeutic potential for this approach.

Periodontal disease is a chronic polymicrobial infectious and inflammatory disease, characterized by the presence of several hundred bacterial species that inhabit the oral cavity and reside within oral biofilms^68–70^. Various mouse models have been proposed in the literature to study human periodontal disease, including ligature models^71,72^, injection models^15^, monomicrobial infection models^73,74^, and polymicrobial infection models^75–77^. Of these, it has been suggested that in polymicrobial infection models, there are synergistic and reciprocal effects on host immunity and physiological responses, and that the pathogenesis of periodontal disease differs from that in monomicrobial infection models^77,78^. In particular, polymicrobial infection models make use of *P. gingivalis*, *T. denticola*, and *T. forsythia* as these are classified as periodontopathic microorganisms and categorized as members of the so called “red complex” because they are strongly associated with clinical parameters of severe periodontal disease, including deep periodontal pocket formation and bleeding on probing^79–82^. Also, *F. nucleatum*, an “orange complex” Gram-negative bacterium that is closely related to the red complex, can aggregate with numerous oral bacteria and can act as an important microbial bridge during biofilm formation^82–84^. In the past, it was believed that the same periodontopathic bacteria that were present in humans were not present in the oral cavity of rodents, but recent comprehensive analyses using PCR arrays and next-generation sequencing have shown that periodontal pathogens, including *P. gingivalis* and *T. forsythia*, are present in rats and mice^67,85^. However, these bacterial strains may differ from the *P.gingivalis* FDC 381 and *T. forsythia* ATCC 43037 strains which were used in this current study. In fact, in this study, even mice without infection exhibited periodontal pathogens but these were different strains from the periodontopathic bacteria administered in the study. Specifically *P. gingivalis* TDC 60 and *T. forsythia* 3313 were detected in all mice, including the control group; indicating that periodontal pathogens are part of the native oral microflora of mice.

These pathogenic bacteria contribute to the pathogenesis of periodontal disease through various mechanisms, including secretion of proteolytic enzymes, host cell invasion, and activation and modulation of host immune responses by LPS and other surface effector molecules^86–89^. For example, in our previous study that utilized a polymicrobial infection model of periodontal disease with the three red complex species (*P. gingivalis*, *T. denticola*, and *T. forsythia*) and *F. nucleatum*, we found an enhanced serum antibody response to these periodontal pathogens, an altered oral microbiome composition, and a corresponding altered cytokine immune response plus alveolar bone resorption characteristic of periodontal disease^57,67^. Therefore, this well characterized polymicrobial infection mouse model that recapitulates the characteristic features of naturally occurring periodontal disease was also used in this study. As expected, our results showed that in the infection group, the compositional changes and decreased diversity in the oral microbiome characterized by an increase in the Proteobacteria and Fusobacteria phylum were accompanied by a significant increase in inflammatory cytokines, an inflammatory cellular infiltrate into the gingival tissues, and alveolar bone resorption (Figs. 1–4). It is interesting that despite high infection levels of periodontopathic bacteria, the only inflammatory cytokines expressed in gingival tissue were IL-6 and CXCL2, which may reflect the “stealth” like properties of *P. gingivalis* as a keystone pathogens. *P. gingivalis* has the ability to evade the host immune response through a cross-talk between the complement system and Toll-like receptors, thereby suppressing the bactericidal action of immune cells and controlling inflammatory responses^2,90^.

The concept that periodontal pathogens induce gut dysbiosis is supported by the fact that the oral microbiome acts as an endogenous reservoir that supplies novel bacteria to the gut microbiota^91^. Humans continuously and unconsciously swallow pathogens present in saliva and dental plaque^41^, and some of those oral bacteria can pass through the harsh acidic environment of the stomach and reach the intestinal tract, even in systemically healthy individuals^43,91^. Several clinical studies have reported marked differences in the composition of the gut microbiome between periodontitis patients and healthy subjects^41–43^. Lourenςo et al.^41^ found that the gut microbiome of patients with chronic periodontitis had a higher abundance of the Firmicutes, Proteobacteria, Verrucomicrobia and Euryarchaeota phylum and a lower abundance of the Bacteroidetes phylum, plus the diversity of the microbiota was decreased. Kawamoto et al.^42^ showed that in fecal samples from patients with severe periodontitis, the Bacteroidia and Actinobacteria phylum exhibited a lower abundance and there was a greater enrichment in several families and genera of the Firmicutes phylum. Furthermore, the microbiomes of periodontitis patients contained a greater abundance of the genus *Acidaminococcus*, *Clostridium*, *Lactobacillus*, *Bifidobacterium*, *Megasphaera*, and *Romboutsiac*. Interestingly, Bao et al.^43^ reported that transplantation of salivary microbiomes from patients with severe periodontitis into wild-type C57BL6 mice increased the proportion of Porphyromonadaceae and Fusobacterium in the gut and concurrently increased the levels of inflammatory cytokines and decreased the expression of tight junction proteins, which are associated with the intestinal barrier in the intestinal epithelium.

Many animal studies have shown that oral administration of periodontal pathogens induces gut dysbiosis and changes in intestinal metabolites, insulin resistance, and hepatic fat deposition in rats and mice^32,43,44,47,92^. For example, *P. gingivalis*-induced gut dysbiosis suppressed the gene expression of tight junction proteins, causing an increase in serum levels of lipopolysaccharide (LPS)^32,47^. Yamazaki et al.^92^ demonstrated that in a mouse model of NAFLD, mice fed a high-fat diet and inoculated with *P. gingivalis* or *P. intermedia* exhibited an altered gut microbiome and blood metabolism, and a shift in hepatic transcriptional expression toward an NAFLD phenotype. In contrast, application of *Actinomyces naeslundii* and *Veillonella rogosae*, which are endemic oral bacteria, had no effect on NAFLD progression. With regard to a polymicrobial infection model, Blasco-Baque et al.^45^ found that in mice fed a high-fat diet, combined administration of *P. gingivalis*, *F. nucleatum*, and *P. intermedia* induced impaired blood glucose metabolism and insulin resistance with concomitant changes in the gut microbial composition. Consistent with these findings, the polymicrobial infection model used in the present study showed marked changes in the composition and diversity of the gut microbiome, inflammation in the intestinal mucosa, and decreased expression of tight junction proteins (Figs. 1–3, 5).

Gut dysbiosis increases intestinal permeability due to the disruption of intercellular junctions of the intestinal mucosa, and increases the translocation of enteric bacteria and their metabolites to the liver via enterohepatic circulation^33,93,94^. Thus, it is likely that in pathological conditions, the liver is constantly exposed to a variety of intestinally-derived substances, including enterobacteria and LPS. Nakajima et al.^47^ reported when the gut microbiota was altered by oral administration of *P. gingivalis*, the gene expression of tight junction proteins decreased in the gut tissues, LPS levels increased in serum, and larger amounts of bacterial DNA were detected in the liver of mice. Similarly, in our study, the total bacterial DNA count in the liver of the infected mice was significantly increased compared to the control mice, and the microbiome composition and diversity in the liver was also markedly different (Figs. 1–3, 6). An increase in Firmicutes and a decrease in Proteobacteria at the phylum level, and an increase in *Lachnospiraceae* at the genus level were characteristic features of the infection group. This indicates that a periodontal polymicrobial infection may increase the bacterial load on the liver through oral and gut dysbiosis. Importantly, a polymicrobial infection markedly increased liver vacuolar degeneration and fat deposition around the central vein in the liver (Fig. 7), similar to previous findings in monomicrobial periodontal infection-induced gut dysbiosis models^32,46,47,92^.

Since gut dysbiosis may cause subsequent fatty liver disease, microbiome-targeted therapeutic approaches using probiotics, prebiotics, and bacteriocins may help prevent the development and progression of NAFLD in patients with periodontal disease. In particular, improving the gut microbiome with probiotics for the treatment of NAFLD has been favorably accepted and supported by many studies^49–51,58^. In preclinical animal studies, probiotics have been shown to ameliorate the increased liver adiposity by suppressing the development of insulin resistance and hepatitis signaling through the regulation of the gut microbiota^53–56^. Randomized controlled trials in NAFLD patients revealed that administration of polymicrobial probiotics (containing *Bifidobacteria*, *Lactobacillus acidophilus*, *L. bulgaricus*, *L. paracasei*, *L. plantarum*, and *Streptococcus thermophilus*) significantly reduced the fatty liver phenotype, inflammation, and fibrosis^53,95,96^.

In this study, in the context of a polymicrobial periodontal infection, oral administration of nisin, a bacteriocin produced primarily by *L. lactis*, shifted the oral, gut, and liver microbiome toward a new state commensurate with health. This prevented periodontal disease and enteritis, and subsequently reduced the bacterial exposure in the liver (Figs. 1–6). In addition, nisin mediated a marked protective effect against vacuolar degeneration and fat deposition in the liver (Fig. 7). In the past, it was believed that nisin exhibited high antimicrobial activity against Gram-positive bacteria but not against Gram-negative bacteria without ethylenediaminetetraacetic acid (EDTA)^97^. However, recent studies have shown that salinity reduces the cell membrane permeability of nisin in Gram-negative bacteria, indicating that nisin alone can have sufficient antimicrobial activity against Gram-negative bacteria when salinity is controlled^98^. Similarly, we have found that nisin has concentration-dependent antimicrobial activity against Gram-negative periodontopathic bacteria such as *P.gingivalis*, *T.denticola*, and *F.nucleatum*^60^. Although nisin can be cleaved by digestive enzymes, it is most stable and soluble at low pH values (pH 2) like that in the gastric environment^99–102^. In this study, nisin was shown to be effective in altering intestinal microbiota and inhibiting intestinal inflammation, thus demonstrating its stability and effectiveness as it traverses low pH and enzymatic environments. Additional explanations for its effectiveness in this mouse model may include: (1) nisin directly changes intestinal bacteria due to a protective effect of the carboxymethyl cellulose (CMC)-Na used as a carrier; (2) The oral flora altered by nisin indirectly shifted the intestinal flora to a different state. It has been reported that 20–30% of endogenous oral bacteria swallowed with saliva reach and colonize the intestine despite the acidic environment in the stomach. In particular, CMC-Na binds to amino acids and proteins via various processes, such as electrostatic interactions, hydrogen bonds, and hydrophobic interactions, which mask the binding site of pepsin^103^. At the same time, CMC-Na is not degraded in the acidic environment of the stomach and becomes insoluble by ionization, but is converted back to a water-soluble sodium salt in the alkaline intestinal stage^104^. Therefore, resistance to digestive enzymes and changes in solvent viscosity and consistency may have reduced nisin digestion.

Limited studies have examined the therapeutic effects of nisin and other bacteriocins on NAFLD, and several in vitro and in vivo studies have been conducted on the effects of oral administration of nisin and nisin-producing *L. lactis* on the digestive tract^105–108^, although none of these studies were performed in the context of periodontal disease. Use of nisin and/or nisin-producing *L. lactis* re-established a balanced gut microbiota and alleviated symptoms compared to conventional antibiotics (vancomycin, metronidazole) and anti-inflammatory drugs (sulfasalazine) in various inflammatory bowel disease mouse models^105–107^. A study by Jia et al.^108^ on the gut-brain axis using a mouse model of *Escherichia coli*-induced diarrhea reported that nisin modulates not only the gut microbiota but also important neurochemicals in the brain and central nervous system, such as norepinephrine, 5-hydroxytryptamine and dopamine. Some in vivo experiments have also reported on the therapeutic effect of *L. lactis* on NAFLD^109–111^. Lee et al.^110^ showed that oral administration of *L. lactis* strain NZ3900 pre-stimulated with nisin significantly limited the formation of a fatty liver phenotype and the progression of early atherosclerosis in a rabbit model fed a high cholesterol diet. Also, Naudin et al.^111^ reported that in mice fed a high-calorie Western diet, oral administration of *L. lactis* subsp cremoris improved glucose tolerance and reduced weight gain, obesity, serum cholesterol levels, and hepatic lipid deposition compared to the beneficial bacteria *Lactobacillus rhamnosus* GGL. In addition, in our study, nisin treatment prevented the increase in Bacteroidetes and decrease in Firmicutes phylum in the polymicrobial infection-induced gut dysbiosis, and improved the bacterial composition to a new state that is also different from that in the control mice (Fig. 1 and Supplementary Fig. 1**)**. This reduction in the Firmicutes/Bacteroidetes ratio is known to be involved in the disturbance of liver glycolipid metabolism and promotion of fatty liver formation in periodontal disease-infected mice^32,92^. Interestingly, at the species level, the abundance of *Lactobacillus gasseri* in the gut and liver microbiomes was increased in the nisin and the infection+nisin groups (Fig. 2). *Lactobacillus* spp. have been known to play a protective role against NAFLD, which may be one explanation for the liver-friendly effects of nisin^112–114^. On the other hand, the microbial composition in the oral cavity of mice treated with nisin alone was closer to that of the infected condition rather than the control condition, which was also reflected in changes in diversity (Fig. 3A, D). In addition, treatment with nisin alone significantly reduced the microbiome diversity in the small intestine (Fig. 3B). These results indicate that nisin itself may induce a new microbial state and that its protective effect may depend on the nature and amount of specific species present in organs.

With respect to bacteria exposed to the liver, it is very interesting that in the context of disease, nisin treatment significantly reduced not only total bacterial counts but also the number of *T.forsythia*. Although *T. forsythia* is known to be a highly pathogenic periodontopathic bacteria detected in the periodontal pockets of patients with severe and refractory periodontal disease, no studies have reported its pathogenicity in liver disease, and this finding may help clarify the relationship between periodontal disease and NAFLD. However, unfortunately, the copy numbers of *P.gingivalis* and *T.forsythia* detected in the liver are low and the possibility that they are background noise cannot be completely excluded.

Mitochondrial dysfunction and oxidative stress may be important mechanistic processes by which nisin attenuates periodontal disease-induced hepatic lipidation and lipid peroxidation. Gene expression analysis in the liver in this study revealed that nisin treatment of polymicrobial-infected mice significantly suppressed the expression of oxidative phosphorylation-related genes in mitochondria and peroxisomes, including cytochrome P450 (Fig. 8, 9). These genes clustered in pathways involved in the pathogenesis of NAFLD, most of which were associated with the mitochondrial electron transfer complex. In general, oxidative stress is defined as a detrimental condition resulting from an imbalance between excessive production of reactive oxygen species (ROS), such as singlet oxygen, superoxide, and hydrogen peroxide, and a lack of antioxidant capacity^115,116^. The major intracellular source of ROS is the mitochondria, and superoxide anion radicals are produced via two main subunits when adenosine triphosphate (ATP) is synthesized through oxidative phosphorylation by the electron transport chain: complex I (NADH dehydrogenase) and complex II (ubiquinone-cytochrome C reductase)^117^. Oxidative stress is strongly involved in the pathogenesis of NAFLD, especially the accumulation of free fatty acids and ROS production in liver tissue, which have been reported to have mutually adverse effects^118–120^. The process by which free radicals pull away electrons from lipids in cell membranes is called lipid peroxidation, resulting in an increase in malondialdehyde (MDA) and 4-hydroxy-2,3-transnonenal (4-HNE), lipid peroxides resulting from cell membranes that cause cell damage and inflammation^121,122^. Lipid peroxides suppress the function of ATP production in the mitochondrial electron transport chain, reduce mitochondrial function through mitochondrial DNA damage and cytochrome c depletion, and further promote ROS production. Indeed, a decreased number and enlargement of mitochondria, increased lipid peroxidation, and reduced ATP levels have been reported in the livers of NAFLD patients^123^.

In the present study, the gene expression of molecules involved in lipid metabolism, PPARα and CYP2E1, was also increased in infected mice, but significantly reduced by nisin treatment. Free fatty acids are known to induce the expression of PPARα, a nuclear receptor-type transcription factor that encodes uncoupling protein-2 (UCP-2), a protein associated with fatty acid oxidation in mitochondria and peroxisomes^124^. Increased expression of UCP-2, a membrane protein present in the mitochondrial inner membrane, impairs oxidative phosphorylation for ATP production. In addition, PPARα also regulates transcription of Acyl-Co-A oxidase, the rate-limiting enzyme for β-oxidation in peroxisomes, and PPARα in hepatic peroxidase is activated in conditions of hepatic lipid accumulation^125^. In other words, increased expression of PPARα contributes to the enhancement of oxidative stress because hydroxyl radicals, the most toxic being ROS, are readily produced during β-oxidation, the process that degrades free fatty acids. In addition, the expression level of CYP2E1, a type of cytochrome P450 enzyme that plays an important role in the metabolism of fatty acids and cholesterol, is increased by free fatty acids and is reported to be significantly higher in the liver tissue of NAFLD patients^126^. CYP2E1 enhances NADPH oxidase activity and superoxide production.

Interestingly, genes involved in iron binding and iron-sulfur cluster binding were increased in the livers of infected mice, but suppressed by nisin treatment (Fig. 10). In contrast, gene expression for metallothionein (Mt) 1, which encodes a metal binding protein important as a metal transporter and antioxidative protein that exerts a metal detoxification function, was significantly decreased in the infection group compared to the control group, and this decrease was prevented by nisin-treatment. Iron, a transition metal element with two oxidation states (divalent and trivalent), catalyzes a reaction in the mitochondrial electron transport chain that converts oxygen to a large number of hydroxyl radicals with strong peroxidative capacity^127,128^. The liver is the largest iron storage organ in humans, and in normal hepatocytes, the majority of iron is stored within the shell of ferritin, an intracellular iron storage protein, and is therefore nontoxic^128^. However, when there is an iron overload in hepatocytes, the increased free iron induces cytotoxicity. An observational study reported by Kowdley et al.^129^ found that intrahepatic iron deposition and hyperferritinemia are frequently observed in patients with NAFLD. Consistent with this result, in the present study, the ferritin gene (Ft) 1 was also significantly increased in the infection group, but this increase was prevented by nisin treatment.

On the other hand, decreases in antioxidant enzymes, such as superoxide dismutase (SOD), catalase, and glutathione have been reported to correlate with the severity of NAFLD^130,131^. SOD is known as an important antioxidant enzyme that converts superoxide to hydrogen peroxide and oxygen, and its active center has zinc, copper and manganese ions as cofactors^128^. Mt1 gene expression, which was markedly decreased by infection but corrected by nisin treatment, is known to bind to these heavy metals and thereby act protectively against oxidative stress and lipotoxicity-induced cellular damage in the liver^132,133^. Hence, as a putative therapeutic mechanism for nisin, Mt1 may counteract periodontal disease-triggered oxidative stress in the liver by alleviating both ROS overproduction and antioxidant deficiencies in this tissue. This is further supported by the fact that nisin significantly prevented the increase in lipid peroxidation markers (MDA) in the liver tissue of the infected mice (Fig. 11).

The prophylactic application of nisin proposed in this study is based on the premise that periodontal disease exacerbates the development and progression of NAFLD in humans. Akinkugbe et al.^20^ demonstrated that there is a causal relationship between periodontal disease and NAFLD and that periodontal disease is a risk for NAFLD based on a population-based prospective cohort study of non-NAFLD subjects. At study entry, subjects were divided into three groups based on the cumulative percentage of periodontal disease present (0%, <30%, and ≥30%), and their liver status was evaluated by ultrasonography and serum ALT after 5 or more years. The results showed that the confounding-adjusted NAFLD incidence rate ratios were statistically significant at 1.28 and 1.60 for the less than 30% and greater than 30% diseased sites, respectively, compared to subjects without periodontal disease. A systematic review by Alakhali et al.^20^ reported a significant correlation between periodontal or bacteriological parameters and NAFLD in all but one of the 12 selected epidemiological studies. In addition, some studies have also found a relationship between NAFLD and tooth loss, the true endpoint of periodontal disease. In this regard, Qiao et al.^134^ used multivariate logistic regression to analyze the association between patients’ self-reported number of missing teeth and NAFLD diagnosed by liver ultrasound in a cross-sectional study of 24,470 Chinese adults, and demonstrated that the number of missing teeth was significantly correlated with the presence of NAFLD in men. Weintraub et al.^135^ also analyzed the relationship between NAFLD, periodontitis, and tooth loss by logistic regression analysis in a population-based cross-sectional study using data from the National Health and Nutrition Examination Survey III in the United States. In a model adjusted for socioeconomic factors, compared to those with good oral health, adults with fewer than 20 teeth or moderate-severe periodontitis had a higher prevalence of NAFLD depending on the measure, such as ultrasonography, Fibrosis Score, and Fatty Liver Index. Consistent with these results, our human autopsy study revealed a negative correlation between the number of remaining teeth and NAFLD tissue scores (Fig. 12). We did not find a correlation with the extent and severity of periodontal disease possibly because measurements of periodontal clinical parameters were not possible in the formalin-fixed cadavers. Therefore the evaluation of periodontal disease was limited to evaluation of Cone beam computed tomography (CBCT) images. Also, although the periodontopathic bacteria that may have mediated the liver damage could not be detected by PCR, they may have exerted their effects earlier on by activating a chronic immune response and triggering epigenetic effects in the liver toward chronic progression of liver disease. However, since the effects of long-term fixation in formalin on bacterial DNA structure and cross-linking reactions cannot be completely eliminated, this may make the analytical results less reliable than those from fresh samples. Furthermore, human autopsy specimens in the present study are from a small sample population with a bias toward the elderly and there is limited data on comorbidities and background information, such as smoking and drinking history. Therefore, there are some limitations in explaining the relationship between periodontal disease and liver disease based on these data alone, and it should be noted that this is only one piece of evidence that supports the higher quality studies reported in the past.

In summary, we have shown that improving oral and gut dysbiosis with nisin lantibiotic treatment is a potential preventive and therapeutic strategy for NAFLD-associated periodontitis. Nisin is safe for daily human consumption, easy to use, and is environmentally friendly (unlike antibiotics whose widespread use has led to contaminated water sources and soils), and can be used alone or in combination with periodontal therapy^24^. In addition, the fact that adjuvant therapy with probiotics for the treatment of periodontal disease is already being tested in clinical applications, this will help facilitate this new treatment strategy^58^. However, this study has some limitations. One limitation is that the gut microbiome dysbiosis was induced not by naturally occurring periodontal disease, but by an oral polymicrobial infection with well-known periodontal pathogens, namely red complex bacteria and *F. nucleatum*. Although we’ve confirmed that periodontal disease develops gradually in this model and it mimics the chronic nature of the disease^57,67^, the composition of the oral bacterial burden that impacts the intestinal tract may not exactly replicate that in naturally occurring periodontal disease in humans. Also, hematogenous diffusion of inflammatory cytokines and periodontal bacteria have been proposed as another pathway by which periodontal disease induces systemic inflammation^15,16,136^, and our study could not completely rule out this effect of periodontitis on the intestine and liver. Nevertheless, given the high prevalence of periodontal disease and the global increase in NAFLD in recent years, this new approach seems extremely important from both a clinical/medical and basic science perspective.

The development of this microbiome-targeted therapy for NAFLD in patients with periodontal disease is still in the early stages. Further studies are warranted to establish the optimal efficacy for host immunomodulatory mechanisms, potential effective combinations of various probiotics and bacteriocins, efficient oral/intestinal delivery methods, and long-term maintenance of microbial composition and functional changes^24,48^. In recent years, there has been concern that highly standardized conditions in a single experiment may reduce the reproducibility of a study, so it may be desirable to introduce a “multicenter” design^137^ or several independent “mini-experiment” designs (conducted at different points during the year within a single institution)^138^ into the design of such a new study. This may improve the reproducibility of animal experiments without increasing sample size. Finally, another notable finding from the sequencing analysis in this study is that polymicrobial oral infections have a significant impact on the liver, far from the site of infection, the periodontal tissue. Consistent and dynamic changes in the liver microbiome, histopathological findings, and gene expression may have critical implications for elucidating the mechanisms by which periodontal disease exacerbates NAFLD.

## Methods

### Ethics approval and Consent to participate

The animal experimental procedures were performed in accordance with the guidelines of Animal Research: Reporting In Vivo Experiments (ARRIVE)^139^ and the guidelines of the Institutional Animal Care and Use Committee of the University of California, San Francisco (IACUC approval number: AN171564-01B). The aspect of the study involving human specimens, including the use of radiographic images was approved by the Human Research Committee of Nippon Dental University (no. NDU-T2021-17).

### Infection and treatment of mice

A total of 24 eight-week old BALB/cByJ female mice (The Jackson Laboratories, Bar Harbor, ME) were housed in microisolator plastic cages at room temperature (23°C) and 50% humidity, with a 12-h light/12-h dark schedule, and fed a standard solid diet. All mice had free access to feed and water from the start of rearing until the end of the experiment.

Mice were randomly distributed into 4 groups (6 mice per group). The description of the experimental groups and infection and treatment protocols are shown in Supplementary Fig. 7. The experimental procedures were approved by the Institutional Animal Care and Use Committee of the University of California, San Francisco (IACUC approval number: AN171564-01B). Please note that due to the need to reduce the number of control animals to uphold best animal use practices, the oral specimens for the animals used in this study were shared with our previous study^57^. To start with, all mice were given trimethoprim (0.17 mg per ml) and sulfamethoxazole (0.87 mg per ml) daily for 7 days in the drinking water and their oral cavity was rinsed with 0.12% chlorhexidine gluconate (Peridex) mouth rinse to inhibit the native oral microbiota as described previously^67^. The polymicrobial inoculum (5×10^9^ combined bacteria per ml; 1×10^9^ cells in 0.2 ml per mouse; 2.5 × 10^8 ^*P. gingivalis*, 2.5 × 10^8 ^*T. denticola*, 2.5 × 10^8 ^*T. forsythia* and 2.5 × 10^8 ^*F. nucleatum*) was then administered topically in the morning for 4 consecutive days every week for a total of 8 weeks. Nisin (300 μg/ml, 0.2 ml per mouse) was administered every day in the evening every week for a total of 8 weeks. A sterile 2% (w/v) CMC (Sigma-Aldrich, St. Louis, MO) solution was administered as the control treatment. Mice were monitored daily for their eating, drinking, activity, and weight to ensure that treatments were well-tolerated.

At 8 weeks following the polymicrobial infection and treatment, oral swab samples were collected from the oral cavity of the mice for assessing the status of the oral microbiome. Changes in the oral cavity due to the polymicrobial infection have been previously reported^57^. The teeth and surrounding gingival tissue were wiped with a sterile cotton swab, and the cotton tip was immersed in 10:1 Tris-EDTA buffer immediately and stored at −80°C until further processing for DNA isolation. Then, mice were humanely euthanized with CO_2_ overdose followed by cervical dislocation per the guidelines recommended by the American Veterinary Medical Association (AVMA). The maxilla and mandibles were resected from each mouse for immunologic and histologic analysis. In addition, the small-intestinal tissue and it’s bowel feces, and liver tissue were collected for histological observation, microbiologic and immunologic assessment by RT-PCR, and sequencing analysis (microbiome and RNA-Sequencing).

### Periodontal bacteria and polymicrobial inoculum

The following periodontal pathogens, namely *P. gingivalis* FDC 381, *T. denticola* ATCC 35405, *T. forsythia* ATCC 43037, and *F. nucleatum* ATCC 10953, were cultured anaerobically (85% N_2_, 10% H_2_, 5% CO_2_) at 37^o^C under anaerobic conditions according to methods described in our previous study^67^. *P. gingivalis and F. nucleatum* were grown for 3 days in Tryptic Soy Broth (Becton Dickinson, Franklin Lakes, NJ) supplemented with 5 mg/ml yeast extract, 0.5 mg/ml L-cysteine hydrochloride, 5 μg/ml hemin, 1 μg/ml menadione and 5% fetal bovine serum (FBS, Gibco Thermo Fisher Scientific, Waltham, MA). *T. denticola* was cultured in Oral Treponeme Enrichment Broth medium (Anaerobe systems, Morgan Hill, CA) for 5 days. *T. forsythia* was grown for 7 days in Tryptic Soy Broth containing 5 mg/ml yeast extract, 0.5 mg/ml L-cysteine hydrochloride, 5 μg/ml hemin, 1 μg/ml menadione, 10 μg/ml *N*-acetylmuramic acid (Sigma-Aldrich, St. Louis, MO), and 5% FBS. Bacterial concentration was determined quantitatively using a spectrophotometer (SpectraMax M2, Molecular Devices, Sunnyvale, CA) and each organism was resuspended in phosphate-buffered saline (PBS) at 1 × 10^10^ bacteria per ml for experiments.

For the oral polymicrobial infection, *P. gingivalis* was mixed with an equal volume of *T. denticola* for 5 min. Subsequently, *T. forsythia* was added to the culture tubes containing *P. Gingivalis* and *T. denticola*, and the bacteria were mixed gently for 1 min and allowed to interact for an additional 5 min. Finally, *F. nucleatum* was added and mixed well with *P. Gingivalis*, *T. denticola*, and *T. forsythia*. After 5 min, the four bacterial consortium was mixed thoroughly with an equal volume of sterile 4% (w/v) carboxymethylcellulose in PBS, and this mixture was used as the polymicrobial oral inoculum. To ensure vitality and viability of the microbes in the polymicrobial inoculum, the bacterial mixture was prepared daily and immediately before each set of innoculations and transported in anaerobic jars for use.

### Nisin preparation

An ultra-pure (>95%) food grade form of nisin Z (NisinZ^®^ P) was purchased from Handary (S.A., Brussels, Belgium), a primary manufacturer of nisin in the food industry. The nisin stock solution was prepared at a concentration of 600 μg/ml in sterile Mili-Q water, filtered using a 0.22 μm syringe filter, and stored at 4°C for a maximum of 5 days for use in experiments^59,67^. For oral treatment of mice, the nisin solution was then mixed with an equal volume of sterile 4% CMC and adjusted to the final concentration of 300 μg/ml.

### DNA isolation from oral swabs, small bowel feces, and liver

DNA from the oral swabs, small bowel feces, and liver was extracted using specific methods for each sample to evaluate microbiological alterations following bacterial challenge and/or nisin treatment by RT-PCR and 16S rRNA sequencing. For the oral swabs and liver tissue, the DNA was isolated and purified using the QIAamp® DNA Mini kit (Qiagen, Hilden, Germany) as in our previous reports^16,67^. Ethanol precipitation of DNA from the oral swabs was further performed to prepare the samples for subsequent analysis. In addition, DNA from the small bowel feces was extracted using the QIAamp® Fast DNA Stool Mini kit (Qiagen) following manufacturer’s protocols. All isolated DNA were stored at -20°C until further processing for real-time PCR and 16S rRNA sequencing analysis.

### RNA isolation from gingival tissue, small intestine, and liver

For RNA isolation, the gingival tissue, small intestine, and liver were treated overnight at 4 °C with RNAlater solution (Invitrogen) immediately after sample collection. Samples were powdered with a mortar and pestle under continuous liquid nitrogen, and total RNA was then isolated from each sample using the Rneasy mini Kit (QIAGEN). The purity and quantity of the RNA were evaluated using the NanoVue Plus spectrophotometer (Biochrom Ltd.). Subsequently, total RNA was synthesized into cDNA with the SuperScript VILO Master Mix (11755050; Invitrogen) following the manufacturer’s protocol.

### Microbiome analysis of oral, small intestine, and liver specimens by 16S rRNA sequencing

The purity and quantity of respective DNA isolated from oral swabs, small bowel feces, and liver were deemed suitable and met quality control measures for 16S rRNA sequencing performed by Novogene, Inc. (en.novogene.com). For the sequencing library preparation, the V4 variable region (515F-806R) of the samples was amplified using specific primers with a barcode. All PCR reactions were carried out with Phusion® High-Fidelity PCR Master Mix (New England Biolabs) and the PCR products were purified with Qiagen Gel Extraction Kit (Qiagen, Germany). The libraries were generated with NEBNext® UltraTM DNA Library Prep Kit for Illumina and were then sequenced by Illumina NovaSeq 6000 System.

Paired-end reads were assigned to samples based on their unique barcodes, truncated by cutting off the barcode and primer sequences, and merged using FLASH (v1.2.7)^140^. Next, quality filtering on the raw tags was performed under specific filtering conditions to obtain the high-quality clean tags according to the analysis pipeline of QIIME (v1.7.0)^141^. Subsequently, the tags were compared with the reference database using UCHIME algorithm^142^ to detect chimera sequences, and the chimera sequences were then removed to obtain the effective Tags. Sequences analysis was performed by Uparse software (v7.0.1001) using all the effective tags. Finally, sequences with ≥97% similarity were assigned to the same operational taxonomic units (OTUs), and species annotation at each taxonomic rank was performed based on comparison to the SSUrRNA database of SILVA Database using Mothur software^143^.

Alpha diversity and the Simpson diversity index were calculated from the number of observed OTUs with QIIME software to evaluate species richness and evenness. Beta diversity analysis was also used to evaluate differences in samples in terms of species complexity. Principal PCoA using weighted UniFrac distance based on I distribution across samples was performed by QIIME to visualize multidimensional data and provide an overview of microbial dynamics in response to polymicrobial infection and nisin treatment. An Analysis of Similarity (ANOSIM) was further performed to determine whether the difference in microbial community structure among groups was significant. When suggested by a positive *R*-value that variation among groups was larger than the variation within groups, the difference among groups was considered significant if the *P* value was less than 0.05. In addition, for the differential abundance analysis of each bacterial taxa, we performed a two-sample *t* test to compute the *p*-values by R software, assuming equal variance in the two groups. The Benjamini and Hochberg procedure was used to correct for multiple comparisons, and the corresponding FDR was further calculated for conducting pair-wise comparisons (e.g., infection versus control). An FDR adjusted *p* value (*q* value) < 0.05 was considered significant.

### Quantification of total bacteria and periodontal pathogens by real-time PCR

Absolute quantification by standard real-time PCR was used to evaluate the abundance of the periodontal pathogens in the oral cavity, small intestine, and liver. Total bacteria and four periodontal pathogens used for the polymicrobial infection were measured by PCR using TaqMan primers and probes (Invitrogen, Supplementary Table 3) corresponding to the 16S rRNA gene. Tenfold serial dilutions of DNA of known concentration were used to construct standard curves for quantification of total bacteria and periodontal pathogens. The amplification was conducted using a QuantStudio 3 Real Time PCR system (Thermo Fisher Scientific) with a final reaction volume of 20 μL that included TaqMan Fast Advanced Master Mix (Applied Biosystems), DNA (15 ng/ μL), primers, and probes. The optimized thermal cycling conditions were as follows: 95 °C for 10 min followed by 50 cycles of denaturing at 95 °C for 15 s, annealing and extension at 60 °C for 1 min. Data were analyzed using QuantStudioTM Design & Analysis Software v1.4.3 (Thermo).

### PCR evaluation of gene expression of gingival tissues and the small intestine

To evaluate immune cytokine profiles from gingival tissues and small intestine, relative gene expression was measured by real-time PCR using the following TaqMan primers and probes (TaqMan Gene Expression Assays; Applied Biosystems): *Il1β* (Mm00434228_m1), *Il4* (Mm00445259_m1), *Il6* (Mm00446190_m1), *Tnf* (Mm00443258_m1), *Ccl2* (Mm00441242_m1), *Cxcl2* (Mm00436450_m1), *Ifng* (Mm01168134_m1) and *Tgfb1* (Mm01178820_m1). Tight junction proteins, which play an important role in gut barrier function, were also analyzed for the small intestine using the following TaqMan primers and probes: *Ocln* (Mm00500912_m1), *Tjp1* (Mm00493699_m1), and *Cldn1* (Mm00516701_m1). Glyceraldehyde 3-phosphate dehydrogenase (*Gapdh*; Mm99999915_g1) was used as a housekeeping gene to normalize the amount of mRNA present in each reaction. PCR was performed in 20 μl reaction mixtures containing the TaqMan Fast Advanced Master Mix, cDNA template (20 ng/μl well), primers, and probes. The optimized thermal cycling conditions were as follows: 20 min at 95°C, followed by 40 cycles per 1 min at 95°C, and 20 min at 60°C. To compare the expression levels among different samples, the relative expression level of the genes was calculated by the comparative CT (ΔΔCT) method using QuantStudioTM Design & Analysis Software.

### Histopathological evaluation of the maxilla and small intestine

The right maxilla and small intestines were resected from each mouse and immediately fixed in 4% paraformaldehyde for 24 h. The maxilla was then decalcified with diethyl pyrocarbonate-treated 0.5 M EDTA (pH 8) for 28 days at room temperature. The specimens were dehydrated and embedded in paraffin using a fully enclosed tissue processor (ASP300S, Leica Biosystems, Buffalo Grove, IL).

The maxilla tissue blocks were cut into serial sections (4 μm) parallel to the mesiodistal plane from the palatal view using a microtome (RM2145; Leica, Hessen), then sections were stained with hematoxylin and eosin (HE; Sigma-Aldrich, St. Louis, MO, USA) for assessment of inflammation. The sections were examined with a stereomicroscope. The number of inflammatory cells within a square field (200 × 200 μm) in the connective tissue adjacent to the gingival epithelium between the first and second molars were counted in three tissue sections per mouse specimen (*n* = 3 per group). These histological analyses were conducted by a skilled examiner in a blinded manner, and all measurements were averaged for each group. Data were expressed as the mean number of cells per 1.0 mm^2^ of connective tissue in the maxillary specimens. In addition, to evaluate alveolar bone resorption, the linear distance from the CEJ to ABC was measured between the maxillary first and second molars.

Serial sections (6 μm) of the small intestine were similarly prepared and stained with HE. Histological scoring of the tissues was performed on three tissue sections per mouse specimen (*n* = 3 per group). Histological inflammatory findings were categorized into five distinct groups based on a previous report^144,145^, and each was graded as follows: grade 0, no sign of inflammation; grade 1, very low-level leukocyte infiltration; grade 2, low- level leukocyte infiltration; grade 3, high-level leukocyte infiltration, high vascular density, and thickened colonic wall; and grade 4, transmural leukocyte infiltration, goblet cell loss, high vascular density, and thickened colonic wall. Data was represented as the mean score value.

### Histopathological analysis of liver tissue by HE and Oil-red staining

The liver tissues were fixed in 4% paraformaldehyde for 24 h then embedded in paraffin or frozen. Paraffin sections (6 μm) were stained with HE as mentioned above. To evaluate fatty depositions in the liver, frozen sections (8 μm) were prepared via a cryostat (CM1950; Leica, Hessen, Germany) and analyzed using Oil red staining. The sections were fixed with 4% paraformaldehyde for 5 min, washed with running tap water for 10 min, and incubated with 60% isopropanol for 5 min. Sections were then stained in the Oil red solution (Sigma-Aldrich, St. Louis, MO) at room temperature for approximately 15 min until the appearance of bright red staining was observed. After washing with 60% isopropanol and distilled water, tissues were counterstained with hematoxylin, washed with distilled water again, and subsequently mounted with ImmunoHistoMount (Sigma). Four different fields (×100 magnification) were randomly selected in Oil red-stained images of three tissue sections per mouse specimen (*n* = 3 per group), and the area of orange-stained fatty deposits was measured using ImageJ analysis software^146^. Data were expressed as the percent of fatty area per 100 μm^2^ in the liver specimens.

### Gene expression profile of liver tissue by RNA sequencing

A total amount of 1 μg RNA extracted from liver tissue per sample was used as the input material for the RNA sample preparations. After purification of mRNA from total RNA using poly-T oligo-attached magnetic beads, sequencing libraries were generated using NEBNext® UltraTM RNA Library Prep Kit for (Illumina®, NEB, USA) following manufacturer’s instructions and index codes were added to attribute sequences to each sample. The clustering of the index-coded samples was performed on a cBot Cluster Generation System using PE Cluster Kit cBot-HS (Illumina). After cluster generation, the library preparations were sequenced on an Illumina platform and 2 × 101-bp paired-end reads were generated. Raw sequencing read data of FASTQ format were firstly processed through fastp. In this step, clean data were obtained from the raw data by removing reads containing adapter and poly-N sequences and reads with low quality. Reference genome and gene model annotation files were downloaded from genome website browser (NCBI/UCSC/Ensembl) directly. Paired-end clean reads were aligned to the mm10 reference genome using the Spliced Transcripts Alignment to a Reference (STAR) software. FeatureCounts was used to count the read numbers mapped of each gene, and then FPKM of each gene was calculated based on the length of the gene and reads count mapped to this gene. DESeq2 R package was used to detect DEGs between two groups. The resulting P values were adjusted by the FDR with the Benjamini and Hochberg’s correction. DEGs were defined when the adjusted *P* value < 0.05 and fold change of FPKM was ≥1.3. The statistical enrichment of DEGs in KEGG (http://www.genome.jp/kegg/) pathways was tested using R package clusterProfiler. Furthermore, GO enrichment analysis (http://www.geneontology.org/) of DEGs was implemented with the annotation dataset for GO biological process and molecular function. KEGG and GO terms with adjusted P value less than 0.05 were considered significantly enrichment.

### Immunohistochemistry of malondialdehyde in liver tissue

Slides containing formalin-fixed paraffin-embedded (FFPE) tissue sections were stained for malondialdehyde using the rabbit-specific horseradish peroxidase (HRP)/3,3’-Diaminobenzidine (DAB) together with the Avidin Biotin Complex Detection immunohistochemistry Kit (Mouse Specific HRP/DAB detection kit, ab64259, Abcam, USA), according to manufacturer’s instructions. Briefly, the slides were deparaffinized by submersing the slides in 100% Xylene (Sigma-Aldrich, USA) for 5 minutes twice, and rehydrated by submersing the slides in ethanol solutions containing increasing percentages of water in solution (100%, 90% and 80%; 5 minutes each). Next, the samples were submersed in a hydrogen peroxide blocking solution (supplied with the kit) for 10 min and washed 2 times in phosphate-buffered saline with 1% Tween 20 (PBS-T). The slides were then submersed in a protein blocking solution (supplied with the kit) and incubated for 5 minutes at room temperature, followed by a PBS-T wash. Next, the samples were immersed in a solution containing 1:250 dilution of anti-malondialdehyde primary antibody (Rabbit polyclonal anti-malondialdehyde antibody, ab27642, Abcam, USA) and incubated for 2 h at room temperature. Then, the slides were washed 3 times in PBS-T and submersed in the biotinylated goat anti-rabbit secondary antibody (supplied with the kit) for 2 hours at room temperature. After washing 3 times in PBS-T, the samples were submersed in a solution containing streptavidin peroxidase (supplied with the kit) and incubated for 10 min at room temperature. Then, the samples were rinsed in PBS-T and a solution containing the DAB Chromogen and its Substrate (diluted to 1x; supplied with the kit) was applied to the samples for 10 min. After rinsing the slides in PBS-T, hematoxylin was added to the slides for 1 min and rinsed in tap water. Finally, the slides were mounted and the samples were imaged in a DM 1000LED Microscope (Leica, Germany).

### Human autopsy study

Japanese human cadavers (*n* = 19) donated by the Department of Anatomy of the Nippon Dental University were used in this study. Based on pre-registration information, subjects with a history of liver disease (alcoholic hepatitis, viral hepatitis, cirrhosis, liver cancer, etc.) and other digestive disorders were excluded from our study. The human cadavers were obtained from a donor-based system using the guidelines included in the Law Concerning Body Donation for Medical and Dental Education (the Body Donation Law) and the Law Concerning Cadaver Dissection and Preservation (LCCDP).

CBCT (AZ 3000CT, Asahi Roentgen Industry, Kyoto, Japan) was used to obtain scanned images of the maxillas and mandibles, including all of the teeth and alveolar bone of the cadavers. The scanning parameters were as follows: the tube voltage was 85 kV, the tube current was 4 mA, the scanning time was 17 seconds, the field of view (FOV) was 79 mm φ × 80 mm H, and the voxel size was 0.155 × 0.155 × 0.155 mm. NEOPREMIUM software (Asahi Roentgen Industry, Kyoto, Japan) was used to generate CBCT images from CBCT data. The number of remaining teeth and the severity of periodontal disease based on alveolar bone resorption were evaluated, and cadavers were then divided into two subgroups: a control non-periodontitis group and a periodontitis group. The periodontitis group had at least one tooth with alveolar bone loss exceeding half the root length in each quadrant of the oral cavity, whereas the control group had no teeth with alveolar bone resorption exceeding ½ the root length.

The gingival and liver tissues were then collected and evaluated for an association between periodontal pathogens and hepatic abnormalities by real-time PCR. To this end, DNA was extracted from these tissues using specific methods (QIAamp DNA FFPE Tissue Kit). Standard real-time PCR was used to detect the presence of periodontal pathogens in the gingival and liver tissues. The 16 S rRNA genes corresponding to total bacteria and four periodontal pathogens were amplified with TaqMan primers and probes (Invitrogen, Supplementary Table 3). The amplification was conducted with a final reaction volume of 20 μL that included TaqMan Fast Advanced Master Mix (Applied Biosystems), DNA (15 ng/ μL), primers, and probes using a StepOnePlus Real Time PCR system (Applied Biosystems). Thermal cycling conditions were as follows: 95 °C for 10 min followed by 50 cycles of denaturing at 95 °C for 15 s, annealing and extension at 60 °C for 1 min. Data were analyzed using StepOnePlus software (Applied Biosystems).

The liver tissue was also fixed with a 20% neutral buffered formalin solution, embedded in paraffin, sectioned (4 μm), and stained with HE. NAFLD activity score (NAS) for the liver specimens was evaluated in the HE stained images in accordance with the definition of Kleiner et al. to diagnose liver disease^147^. Total NAS in individual subjects was calculated as the sum of three scores, including steatosis, inflammation, and cell injury (ballooning).

### Statistical analysis

A power analysis was performed to determine the optimal sample size for the present animal study based on data from our previous study,^67^ relative to the mean difference and SD for the level of IL-6 gene expression using G*Power 3 analysis software (Heinrich-Heine-University Dusseldorf, Düsseldorf, Germany). The required minimum sample size of mice was determined as 5 to obtain a power of 80% with α = 0.05. Although 6 mice were included in each group to account for accidental animal deaths. Measurements for all assays were taken from distinct samples. All evaluations were carried out by one calibrated and blinded examiner. SPSS 21.0 statistical software (IBM, Chicago, IL, USA) was used for statistical analysis of the non-sequencing data. The analysis of the bacterial number and immune profile-related gene expression, and inflammatory cell infiltrate in the gingival tissue were compared using ANOVA and Tukey’s test for multiple comparison among 4 groups. Student’s *t* test was used to compare values between two groups. The histological score for enteritis and lipid deposition area in the liver were analyzed by a Kruskal–Wallis H-test followed by a Steel-Dwass test or Dunn’s test for nonparametric data. For the human autopsy study, the Pearson correlation coefficient was used to analyze the correlation between the number of remaining teeth and NAS, and the required sample size was calculated as 18 to obtain a power of 80% with α = 0.05 using G*Power 3 for effect size of 0.58. A *p* value less than 0.05 was considered to be significant.

### Reporting summary

Further information on research design is available in the Nature Research Reporting Summary linked to this article.

### Supplementary information

Supplementary information

Reporting Summary

## Data Availability

The data that support the findings of this study are openly available in figshare at 10.6084/m9.figshare.22144736.

## Code availability

The codes that support the findings of this study are openly available in figshare at 10.6084/m9.figshare.22144736.
