# Supplementary material for: Nisin lantibiotic prevents NAFLD liver steatosis and mitochondrial oxidative stress following periodontal disease by abrogating oral, gut and liver dysbiosis
Source: NPJ Biofilms Microbiomes. 2024 Jan 17;10:3. doi: 10.1038/s41522-024-00476-x (PMC10794237; doi:10.1038/s41522-024-00476-x)
Supplement: Supplementary file 1 — Supplementary information [file 41522_2024_476_MOESM1_ESM.pdf]

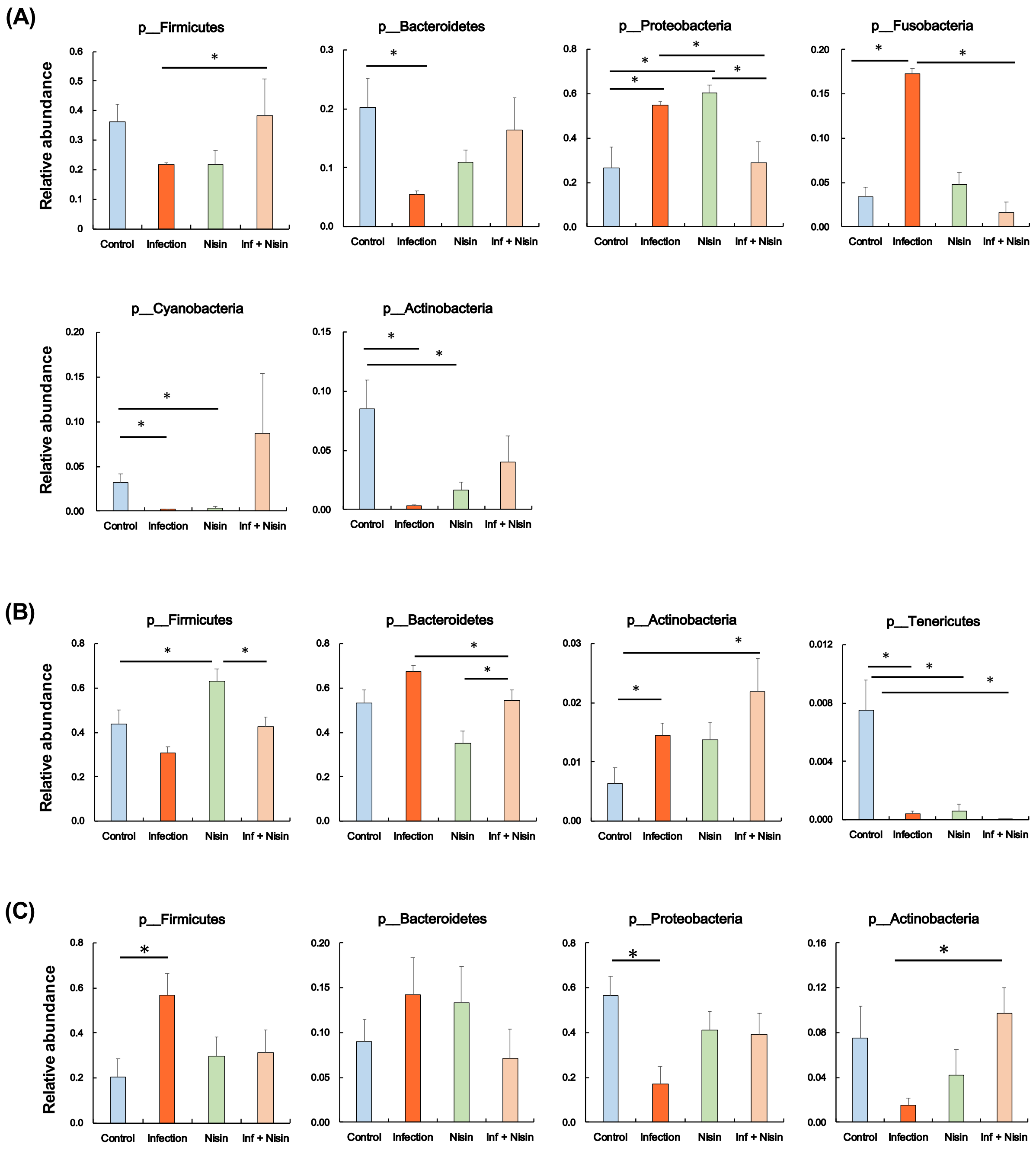

Supplementary Figure 1.
Between-group variation analysis of bacteria taxa at the phylum level in the oral **(A)**, gut **(B)** and liver **(C)**. The data in the bar graphs are shown as means ± standard deviation. *$p < 0.05$ between groups with Tukey test (n=6).

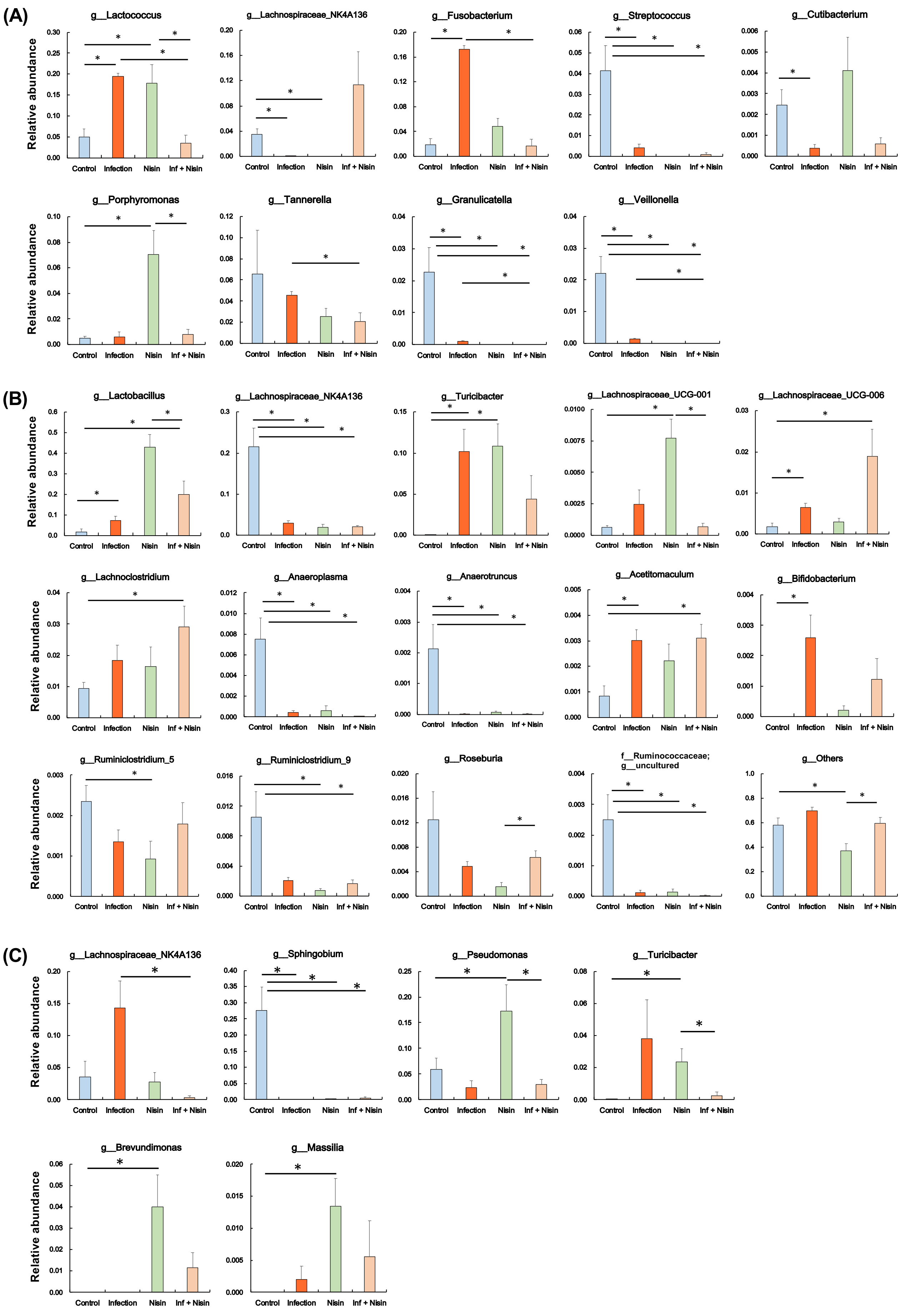

Supplementary Figure 2. Between-group variation analysis of bacteria taxa at the genus level in the oral **(A)**, gut **(B)** and liver **(C)**. The data in the bar graphs are shown as means ± standard deviation. *$p < 0.05$ between groups with Tukey test (n=6).

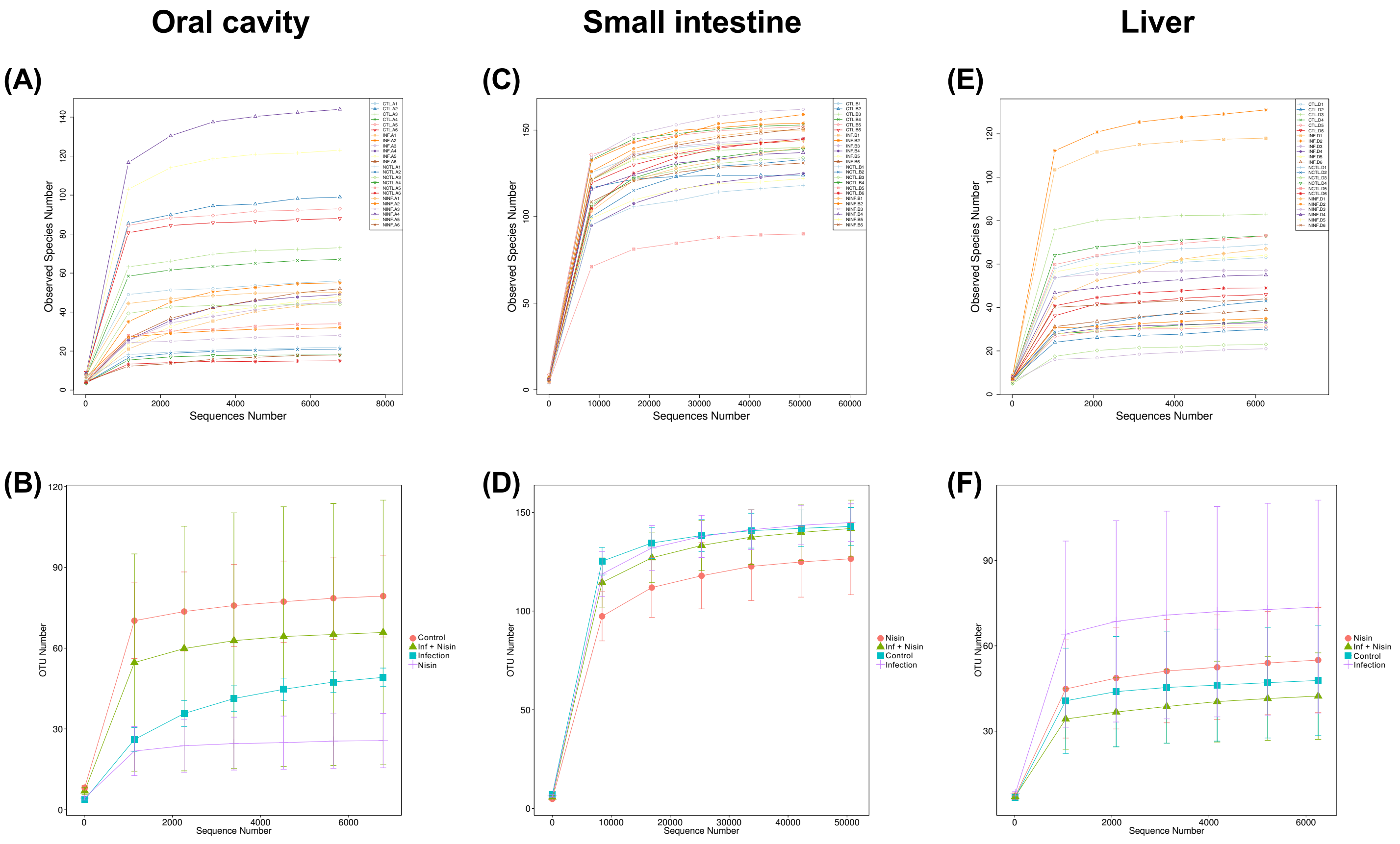

Supplementary Figure 3.

Rarefaction curves of each sample **(A, C, and E)** and group**(B, D, and F)** in the oral cavity, small intestine, and liver (n=6).

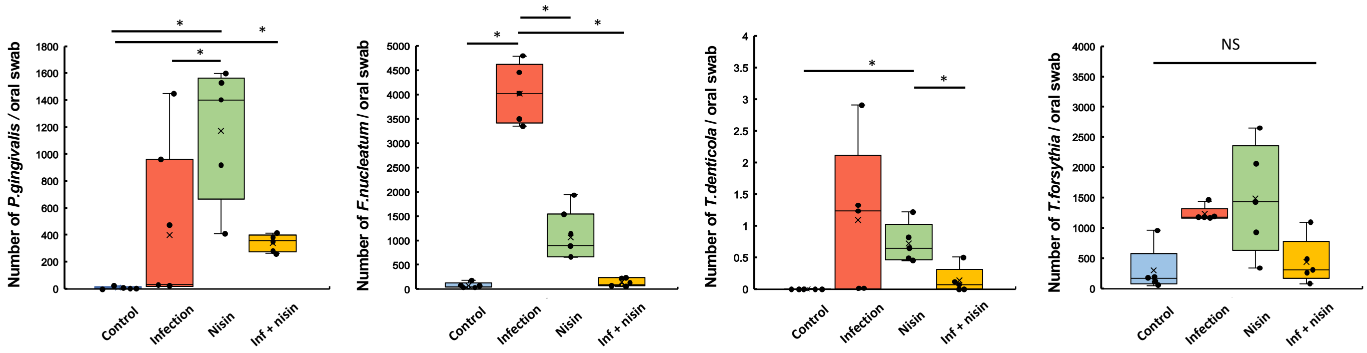

Supplementary Figure 4.
The number of periodontal pathogens in oral cavity following polymicrobial infection and nisin treatment. Box-plots display the median, first quartiles (25th percentile), third quartiles (75th percentile), and minimum and maximum whiskers, with all data points plotted. *p < 0.05 between groups with Tukey test (n=5).

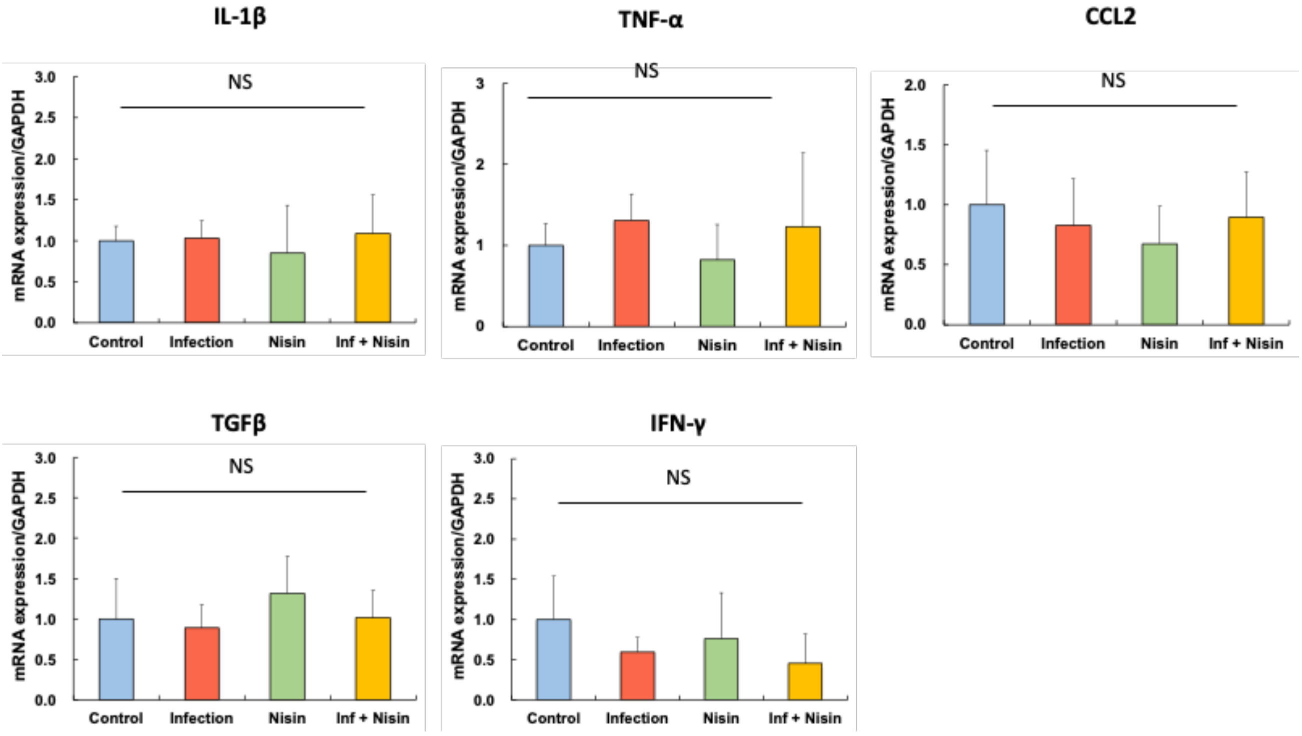

Supplementary Figure 5.

Gene Expression of Pro-inflammatory Cytokine in Periodontal Tissue.

The data in the bar graphs are shown as means ± standard deviation.

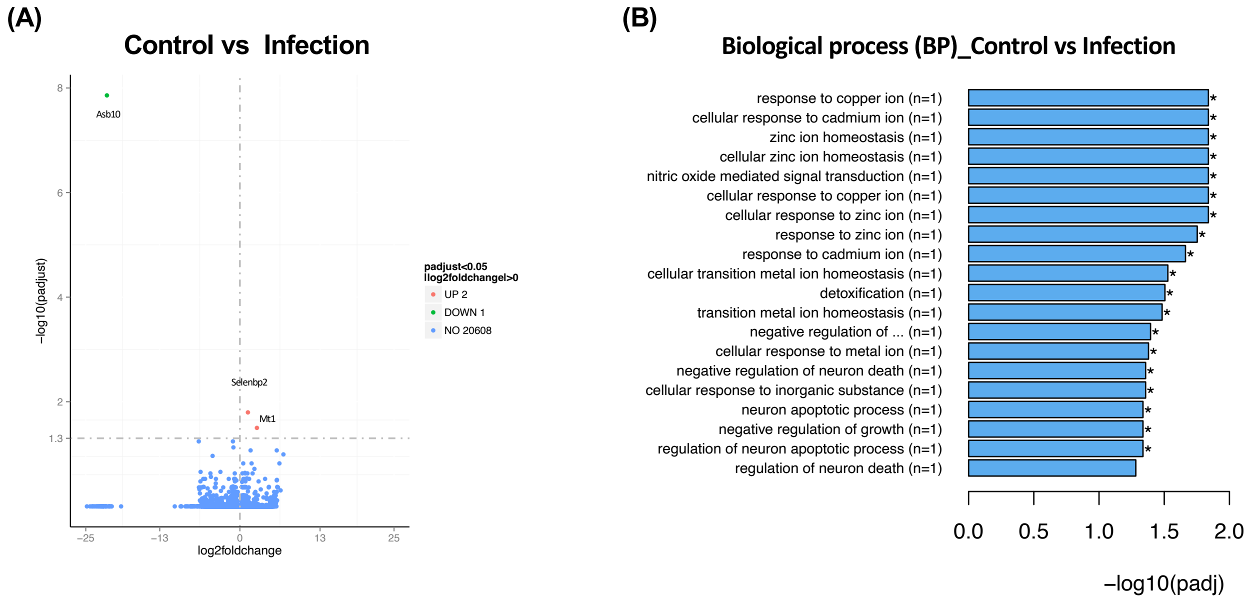

Supplementary Figure 6.

Gene profile in liver tissue analyzed by RNA sequencing (n=6). **A)** Volcano plot revealed 3 DEGs (2 up-regulated and 1 down-regulated) between the control group and the infection group. **B**) Bar plot shows function of expressed genes for top 20 significantly enriched GO terms at biological process category, which were determined by ClusterProfiler (version 3.8.1).

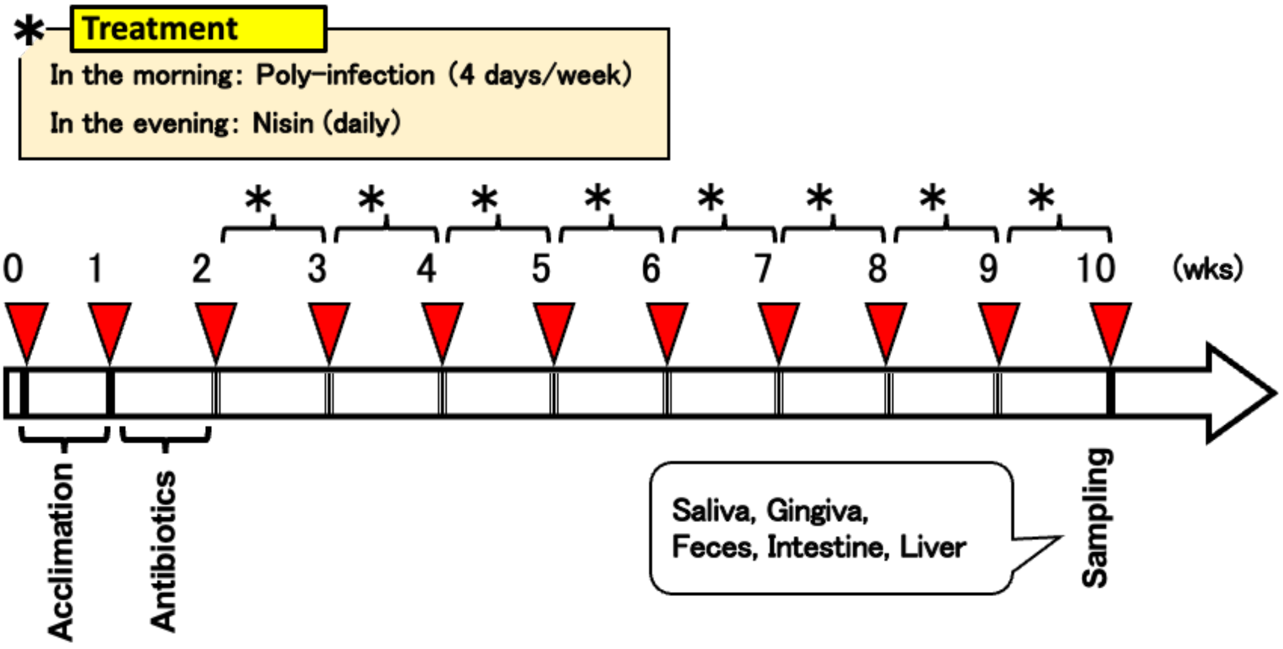

Supplementary Figure 7. Experimental design using polymicrobial infection animal model

Supplementary Table 1. Microbial genera identified via 16S sequencing in the oral cavity, small intestine and liver; ranked by their relative abundance (from highest at the top to lowest at the bottom).

Oral	Small intestine	Liver
Massilia	*Lactobacillus*	*Sphingobium*
Lactococcus	*Lachnospiraceae_NK4A136_group*	*Lactobacillus*
Lachnospiraceae_NK4A136_group	*Turicibacter*	*Pseudomonas*
Tannerella	*Alistipes*	*Lachnospiraceae_NK4A136_group*
Fusobacterium	*Lachnoclostridium*	*Novosphingobium*
Porphyromonas	*Lachnospiraceae_UCG-006*	*Sphingomonas*
Anaerococcus	*Enterorhabdus*	*Anoxybacillus*
Staphylococcus	*Candidatus_Saccharimonas*	*Thermicanus*
Brevundimonas	*Intestinimonas*	*Thermus*
Shimwellia	*Enterobacter*	*Collinsella*
Pigmentiphaga	*uncultured Lachnospiracea*	*Corynebacterium_1*
Dyadobacter	*Roseburia*	*Turicibacter*
Streptococcus	*Lachnospiraceae_A2*	*Granulicatella*
Corynebacterium_1	*Ruminiclostridium*	*Staphylococcus*
Pedobacter	*Ruminiclostridium_9*	*Lachnospiraceae_UCG-006*
Allorhizobium-Neorhizobium-Pararhizobium-Rhizobium	*Oscillibacter*	*Methyloversatilis*
Hymenobacter	*Lachnospiraceae_ASF356*	*Alistipes*
Anaeroplasma	*Ruminococcaceae_UCG-014*	*Stenotrophomonas*
Pseudomonas	*Blautia*	*Prevotella_7*
Neisseria	*[Eubacterium]_xylanophilum_group*	*Bradyrhizobium*
Phytophthora_lateralis_MPF4	*Clostridium_sensu_stricto_1*	*Alkanindiges*
Lactobacillus	*Anaeroplasma*	*Brevundimonas*
Prevotellaceae_UCG-001	*Lachnospiraceae_UCG-001*	*Tepidiphilus*
Atopobium	*GCA-900066575*	*uncultured Caldilineaceae*
Roseburia	*Candidatus_Arthromitus*	*uncultured Lachnospiraceae*
uncultured Lachnospiraceae	*Tyzzerella_3*	*[Eubacterium]_xylanophilum_group*
Megasphaera	*Enterococcus*	*Enterorhabdus*
Granulicatella	*Anaerotruncus*	*Hydrogenophaga*
uncultured_Bacteroidales_bacterium	*Acetitomaculum*	*Acinetobacter*
Mogibacterium	*Stenotrophomonas*	*Paenibacillus*
Prevotella_7	*uncultured Ruminococcaceae*	*Prevotella_6*
Leptotrichia	*uncultured Peptococcaceae*	*Dechloromonas*
Aeromicrobium	*Bifidobacterium*	*Candidatus_Alysiosphaera*
Bacteroides	*Ruminiclostridium_5*	*Flavobacterium*

(Continues)

Supplementary Table 1

Veillonella	*Ruminiclostridium_6*	*Burkholderia-Caballeronia-Paraburkholderia*
Peptoniphilus	*Bacteroides*	*Ruminiclostridium_9*
Noviherbaspirillum	*Streptococcus*	*Aeromonas*
Tepidimonas	*Acetatifactor*	*Roseburia*
Intestinimonas	*Prevotellaceae_UCG-001*	*Lachnospiraceae_GCA-900066575*
Lawsonella	*Parvibacter*	*Cloacibacterium*
Lachnospiraceae_UCG-006	*Ruminococcaceae_UCG-009*	*Peptoniphilus*
Alloprevotella	*Cenchrus_americanus*	*Lactococcus*
Alistipes	*Sphingomonas*	*Streptococcus*
Devosia	*Rhodobacter*	*Rothia*
Corynebacterium	*Lachnospiraceae_FCS020_group*	*Lachnoclostridium*
Methyloversatilis	*Pseudomonas*	*Ruminococcaceae_UCG-014*
Youngiibacter	*Methylobacterium*	*Methylomonaceae_pLW-20*
Sphingomonas	*Harryflintia*	*Massilia*
Haemophilus	*[Eubacterium]_brachy_group*	*Sporichthyaceae_hgcI_clade*
Micrococcus	*Marvinbryantia*	*Kocuria*
Ruminococcaceae_UCG-014	*Ruminococcaceae_UCG-010*	*Ruminococcus_1*
Escherichia-Shigella	*Alloprevotella*	*Ruminococcaceae_UCG-004*
uncultured Caulobacteraceae	*Others*	*Methylobacterium*
Actinomyces		*Bacillus*
Caulobacter		*Rickettsiella*
Methylobacterium		*Cutibacterium*
Nocardioides		*Veillonella*
Lachnoclostridium		*Anaerococcus*
Rhodococcus		*Lachnospiraceae_A2*
Enterorhabdus		*Ruminiclostridium_5*
[Eubacterium]_xylanophilumgroup		*Sediminibacterium*
uncultured Veillonellaceae		*Anaeroplasma*
Prevotella_6		*uncultured Diplorickettsiaceae*
Rothia		*Roseomonas*
Chryseobacterium		*Neisseria*
Cetobacterium		*Pantoea*
Gemella		*Micrococcus*
uncultured Neisseriaceae		*Megamonas*
Stomatobaculum		*Limnohabitans*
Thermicanus		*Alloprevotella*
Ruminiclostridium		*uncultured Chitinophagaceae*
Marvinbryantia		*Micromonospora*

(Continues)

Supplementary Table 1

Brachybacterium		*uncultured Peptococcaceae*
Cytophaga		*Lachnospiraceae_UCG-001*
Flavobacterium		*Pseudoxanthomonas*
uncultured Cyclobacteriaceae		*Romboutsia*
Lachnospiraceae_A2		*Gaiella*
Oscillibacter		*Candidatus_Saccharimonas*
Enterococcus		*Enhydrobacter*
Cutibacterium		*Fodinicola*
Stenotrophomonas		*Leptotrichia*
TM7_phylum_sp._oral_clone_FR058		*Haemophilus*
Ilumatobacteraceae_CL500-29_marine_group		*Caulobacter*
Solobacterium		*Lautropia*
Actinocatenispora		*Corynebacterium*
Ralstonia		*Acetitomaculum*
Blautia		*Bosea*
Belnapia		*Enterococcus*
Novosphingobium		*Porphyromonas*
Acetatifactor		*Blautia*
Prevotella		*Chryseobacterium*
Hyphomicrobium		*[Eubacterium]_coprostanoligenes_group*
Acetitomaculum		*uncultured Veillonellaceae*
uncultured Rhizobiaceae		*Lawsonella*
Salinicoccus		*Fastidiosipila*
Lachnospiraceae_UCG-001		*Geobacillus*
Vibrionimonas		*uncultured Neisseriaceae*
Ruminiclostridium_5		*Conexibacter*
Brevibacterium		*Paracoccus*
[Eubacterium]_coprostanoligenes_group		*uncultured Ruminococcaceae*
Georgenia		*Actinotalea*
uncultured_Clostridiales_bacterium		*Proteiniphilum*
Truepera		*Ruminiclostridium*
Aegilops_tauschii		*Acetobacterium*
Peptostreptococcus		*Butyrivibrio*
Lachnospiraceae_ASF356		*Ilumatobacteraceae_CL500-29_marine_group*
Paenibacillus		*Phytophthora_lateralis_MPF4*
Brevibacillus		*Pedobacter*
Catonella		*Prevotella_9*

(Continues)

Supplementary Table 1

Lachnospiraceae_GCA-900066575		*uncultured_Anaerolineaceae_bacterium*
Geobacillus		*Undibacterium*
Thioclava		*Lachnospiraceae_ASF356*
Blastococcus		*uncultured Sandaracinaceae*
Turicibacter		*Ruminococcaceae_UCG-013*
Limnohabitans		*Ruminiclostridium_6*
Thermomonas		*uncultured Verrucomicrobiaceae*
Parvimonas		*Marmoricola*
Rhodocyclaceae_C39		*Christensenellaceae_R-7_group*
Ruminococcus_1		*uncultured Microscillaceae*
Phreatobacter		*Spirochaetaceae_GWE2-31-10*
Acinetobacter		*Prevotella*
Fodinicola		*Tepidimonas*
Bacillus		*Prevotella_2*
Porphyrobacter		*Nocardioides*
Eikenella		*Leptospira*
Pantoea		*Lachnospiraceae_FCS020_group*
Treponema_2		*Oribacterium*
Spirosoma		*Actinomyces*
Medicago_truncatula_(barrel_medic)		*Thauera*
Curvibacter		*Anaerotruncus*
Capnocytophaga		*Atopobium*
Lachnospiraceae_FCS020_group		*Weissella*
uncultured Ruminococcaceae		*Bacteriovorax*
Psychrobacter		*Polyangium*
Tetrasphaera		*Shuttleworthia*
Alloscardovia		*Candidatus_Firestonebacteria_bacterium_RIFOXYA2_FULL_40_8*
Tyzzerella_3		*Dyella*
uncultured Peptococcaceae		*uncultured_Bacteriovorax_sp.*
Ruminiclostridium_6		*uncultured Erysipelotrichaceae*
Lachnoanaerobaculum		*Clostridium_sensu_stricto_1*
Deinococcus		*Ruminococcaceae_UCG-009*
Family_XIII_AD3011_group		*Oscillibacter*
Cloacibacterium		*Marvinbryantia*
Anoxybacillus		*groundwater_metagenome*
Tyzzerella		*Stomatobaculum*
Anaerotruncus		*Allorhizobium-Neorhizobium-Pararhizobium-Rhizobium*

(Continues)

Supplementary Table 1

[Eubacterium]_nodatum_group		*Ruminococcaceae_UCG-010*
Butyricicoccus		*Eikenella*
Parvibacter		*Ruminococcaceae GCA-900066225*
Family_XIII_UCG-001		*Harryflintia*
Ruminococcaceae_NK4A214_group		*Escherichia-Shigella*
Ruminococcaceae_UCG-010		*Blastococcus*
[Eubacterium]_brachy_group		*Streptomyces*
Campylobacter		*Others*
Ruminococcaceae_UCG-004		
Polynucleobacter		
Paenalcaligenes		
Prevotellaceae_UCG-003		
Murdochiella		
Thermovirga		
Others		

Supplementary Table 2. Individual characteristics of the cadavers.

Age	Sex	No of remaining teeth (maxilla/mandible)	Cause of death	Other underlying diseases
80	Female	12 (4/8)	Senility	
80	Female	13 (7/6)	Transverse colon cancer	
85	Female	15 (7/8)	Pancreatic cancer	
85	Male	12 (12/0)	Lung cancer	
86	Male	29 (15/14)	Senility	
86	Male	26 (12/14)	Senility	Transverse colon cancer
86	Male	22 (9/13)	Acute exacerbation of chronic renal failure	
89	Male	25 (13/12)	Pneumonia	
91	Male	21 (9/12)	Senility	
92	Female	25 (13/12)	Brain stem hemorrhage	High blood pressure
94	Male	26 (14/12)	Lethal irregular veins	
99	Female	27 (13/14)	Senility	Alzheimer's disease and advanced gastric cancer

Supplementary Table 3. PCR primers and probe used for periodontal pathogens and total bacteria.

Bacterium	Sequence (5’-3’)
Porphyromonas gingivalis	F: TAGCTTGCTAAGGTCGATGG
	R: CAAGTGTATGCGGTTTTAGT
	P: [6-FAM] TGCGTAACGCGTATGCAACTTGCC [TAMRA]
Fusobacterium nucleatum	F: CGCAGAAGGTGAAAGTCCTGTAT
	R: TGGTCCTCACTGATTCACACAGA
	P: [6-FAM] ACTTTGCTCCCAAGTAACATGGAACACGAG [TAMRA]
Treponema denticola	F: TAAGGGCGGCTTGAAATAATAATG
	R: AGAGCAAGCTCTCCCTTACCGT
	P: [6-FAM] CAGCGTTCGTTCTGAGCCAGGATCA [TAMRA]
Tannerella forsythia	F: CGACGGAGAGTGAGAGCTTTCT
	R: GCGCTCGTTATGGCACTTAAG
	P: [6-FAM] CGTCTATGTAGGTGCTGCATGGTTGTCG [TAMRA]
Universal (total bacteria)	F: TGCGGGACTTAACCCAACA
	R: TGGAGCATGTGGTTTAATTCGA
	P: [6-FAM] CACGAGCTGACGACARC* [TAMRA]

F, forward primer; R, reverse primer; P, Taqman probe

* "R" indicates mixed sequence of A and G bases.
