# Supplementary material for: Nisin lantibiotic prevents NAFLD liver steatosis and mitochondrial oxidative stress following periodontal disease by abrogating oral, gut and liver dysbiosis
Source: NPJ Biofilms Microbiomes. 2024 Jan 17;10:3. doi: 10.1038/s41522-024-00476-x (PMC10794237; doi:10.1038/s41522-024-00476-x)
Supplement: Supplementary file 2 — Reporting Summary [file 41522_2024_476_MOESM2_ESM.pdf]

nature portfolio

Corresponding author(s): Yvonne Kapila

Last updated by author(s): Nov 7, 2023

Reporting Summary

Nature Portfolio wishes to improve the reproducibility of the work that we publish. This form provides structure for consistency and transparency in reporting. For further information on Nature Portfolio policies, see our Editorial Policies and the Editorial Policy Checklist.

Statistics

For all statistical analyses, confirm that the following items are present in the figure legend, table legend, main text, or Methods section.

n/a	Confirmed	
☐	☒	The exact sample size (*n*) for each experimental group/condition, given as a discrete number and unit of measurement
☐	☒	A statement on whether measurements were taken from distinct samples or whether the same sample was measured repeatedly
☐	☒	The statistical test(s) used AND whether they are one- or two-sided<br>*Only common tests should be described solely by name; describe more complex techniques in the Methods section.*
☒	☐	A description of all covariates tested
☒	☐	A description of any assumptions or corrections, such as tests of normality and adjustment for multiple comparisons
☐	☒	A full description of the statistical parameters including central tendency (e.g. means) or other basic estimates (e.g. regression coefficient) AND variation (e.g. standard deviation) or associated estimates of uncertainty (e.g. confidence intervals)
☐	☒	For null hypothesis testing, the test statistic (e.g. *F*, *t*, *r*) with confidence intervals, effect sizes, degrees of freedom and *P* value noted<br>*Give P values as exact values whenever suitable.*
☒	☐	For Bayesian analysis, information on the choice of priors and Markov chain Monte Carlo settings
☒	☐	For hierarchical and complex designs, identification of the appropriate level for tests and full reporting of outcomes
☐	☒	Estimates of effect sizes (e.g. Cohen's *d*, Pearson's *r*), indicating how they were calculated

Our web collection on statistics for biologists contains articles on many of the points above.

Software and code

Policy information about availability of computer code

Data collection	DNA (microbiome) libraries were sequenced by Illumina NovaSeq 6000 System.<br>RNA sequencing libraries were generated using NEBNext® UltraTM RNA Library Prep Kit for (Illumina®, NEB, USA)
Data analysis	SPSS 21.0 statistical software (IBM, Chicago, IL, USA) was used for statistical analysis of the non-sequencing data.<br><br>For microbiome/DNA sequencing data: Paired-end reads were assigned to samples based on their unique barcodes, truncated by cutting off the barcode and primer sequences, and merged using FLASH (v1.2.7) 68. Next, quality filtering on the raw tags was performed under specific filtering conditions to obtain the high-quality clean tags according to the analysis pipeline of QIIME (v1.7.0) 69. Subsequently, the tags were compared with the reference database using UCHIME algorithm 70 to detect chimera sequences, and the chimera sequences were then removed to obtain the effective Tags. Sequences analysis was performed by Uparse software (v7.0.1001) using all the effective tags. Finally, sequences with ≥97% similarity were assigned to the same operational taxonomic units (OTUs), and species annotation at each taxonomic rank was performed based on comparison to the SSUrRNA database of SILVA Database using Mothur software 71. Alpha diversity and the Simpson diversity index were calculated from the number of observed OTUs with QIIME software to evaluate species richness and evenness.<br><br>RT-PCR data were analyzed using QuantStudioTM Design & Analysis Software v1.4.3 (Thermo).<br><br>For RNA Sequencing: sequencing libraries were generated using NEBNext® UltraTM RNA Library Prep Kit for (Illumina®, NEB, USA) following manufacturer's instructions and index codes were added to attribute sequences to each sample. The clustering of the index-coded samples was performed on a cBot Cluster Generation System using PE Cluster Kit cBot-HS (Illumina). After cluster generation, the library preparations were sequenced on an Illumina platform and 2 × 101-bp paired-end reads were generated. Raw sequencing read data of FASTQ format were firstly processed through fastp. In this step, clean data were obtained from the raw data by removing reads containing adapter and poly-N

sequences and reads with low quality. Reference genome and gene model annotation files were downloaded from genome website browser (NCBI/UCSC/Ensembl) directly. Paired-end clean reads were aligned to the mm10 reference genome using the Spliced Transcripts Alignment to a Reference (STAR) software. FeatureCounts was used to count the read numbers mapped of each gene, and then FPKM of each gene was calculated based on the length of the gene and reads count mapped to this gene. DESeq2 R package was used to detect differentially expressed genes (DEGs) between two groups. The resulting P values were adjusted by the FDR with the Benjamini and Hochberg's correction. DEGs were defined when the adjusted P value < 0.05 and fold change of FPKM was ≥1.3. The statistical enrichment of DEGs in Kyoto Encyclopedia of Genes and Genomes (KEGG, http://www.genome.jp/kegg/) pathways was tested using R package clusterProfiler. Furthermore, Gene Ontology (GO, http://www.geneontology.org/) enrichment analysis of DEGs was implemented with the annotation dataset for GO biological process and molecular function. KEGG and GO terms with adjusted P value less than 0.05 were considered significantly enrichment.

For manuscripts utilizing custom algorithms or software that are central to the research but not yet described in published literature, software must be made available to editors and reviewers. We strongly encourage code deposition in a community repository (e.g. GitHub). See the Nature Portfolio guidelines for submitting code & software for further information.

Data

Policy information about availability of data

All manuscripts must include a data availability statement. This statement should provide the following information, where applicable:

- Accession codes, unique identifiers, or web links for publicly available datasets
- A description of any restrictions on data availability
- For clinical datasets or third party data, please ensure that the statement adheres to our policy

The data and code that support the findings of this study are openly available in figshare at https://doi.org/10.6084/m9.figshare.22144736

Research involving human participants, their data, or biological material

Policy information about studies with human participants or human data. See also policy information about sex, gender (identity/presentation), and sexual orientation and race, ethnicity and racism.

Reporting on sex and gender	Female mice were used for the study.<br>Human cadavers were from both males and females
Reporting on race, ethnicity, or other socially relevant groupings	Human cadavers were all Japanese
Population characteristics	Age and diagnosis at cause of death were repported
Recruitment	Human samples were pilot samples of convenience that were dontated
Ethics oversight	The study was approved by the Human Research Committee of Nippon Dental University (no. NDU-T2021-17). The human cadavers were obtained from a donor-based system using the guidelines included in the Law Concerning Body Donation for Medical and Dental Education (the Body Donation Law) and the Law Concerning Cadaver Dissection and Preservation (LCCDP).

Note that full information on the approval of the study protocol must also be provided in the manuscript.

Field-specific reporting

Please select the one below that is the best fit for your research. If you are not sure, read the appropriate sections before making your selection.

☒ Life sciences ☐ Behavioural & social sciences ☐ Ecological, evolutionary & environmental sciences

For a reference copy of the document with all sections, see nature.com/documents/nr-reporting-summary-flat.pdf

Life sciences study design

All studies must disclose on these points even when the disclosure is negative.

Sample size	A power analysis was performed to determine the optimal samples size for the animal studies. The required minimum sample size of mice was determined as 5 to obtain a power of 80% with α = .05. Although 6 mice were included in each group to account for accidental animal deaths. The human samples were a convenience/pilot set of samples.
Data exclusions	No data were excluded from the analyses.
Replication	All experiments had replicates as noted in each figure legend.
Randomization	Mice were randomly assigned into four treatment groups.

Blinding	Sequencing and its analyses and histological analyses were conducted by skilled examiners in a blinded manner.

Reporting for specific materials, systems and methods

We require information from authors about some types of materials, experimental systems and methods used in many studies. Here, indicate whether each material, system or method listed is relevant to your study. If you are not sure if a list item applies to your research, read the appropriate section before selecting a response.

Materials & experimental systems

n/a	Involved in the study
☐	☒ Antibodies
☒	☐ Eukaryotic cell lines
☒	☐ Palaeontology and archaeology
☐	☒ Animals and other organisms
☐	☒ Clinical data
☒	☐ Dual use research of concern
☒	☐ Plants

Methods

n/a	Involved in the study
☒	☐ ChIP-seq
☒	☐ Flow cytometry
☒	☐ MRI-based neuroimaging

Antibodies

Antibodies used	rabbit anti-malondialdehyde primary antibody (ab243066; Abcam, USA) and mouse specific HRP/DAB Detection IHC Kit (ab64259); both from Abcam
Validation	The rabbit anti-malondialdehyde primary antibody has been validated in mice by numerous publications as noted by the Abcam manufacturer's website: https://www.abcam.com/products/primary-antibodies/malondialdehyde-antibody-11e3-ab243066.html<br><br>The mouse specific HRP/DAB (ABC) Detection IHC Kit (ab64259) has been validated by Abcam: https://www.abcam.com/products/ihc-kits/mouse-specific-hrpdab-abc-detection-ihc-kit-ab64259.html

Animals and other research organisms

Policy information about studies involving animals; ARRIVE guidelines recommended for reporting animal research, and Sex and Gender in Research

Laboratory animals	24 eight-week old BALB/cByJ female mice were purchased from The Jackson Laboratories, Bar Harbor, ME
Wild animals	*Provide details on animals observed in or captured in the field; report species and age where possible. Describe how animals were caught and transported and what happened to captive animals after the study (if killed, explain why and describe method; if released, say where and when) OR state that the study did not involve wild animals.*
Reporting on sex	Only female mice were used
Field-collected samples	*For laboratory work with field-collected samples, describe all relevant parameters such as housing, maintenance, temperature, photoperiod and end-of-experiment protocol OR state that the study did not involve samples collected from the field.*
Ethics oversight	The experimental procedures were approved by the Institutional Animal Care and Use Committee of the University of California, San Francisco (IACUC approval number: AN171564-01B).

Note that full information on the approval of the study protocol must also be provided in the manuscript.

Clinical data

Policy information about clinical studies

All manuscripts should comply with the ICMJE guidelines for publication of clinical research and a completed CONSORT checklist must be included with all submissions.

Clinical trial registration	*Provide the trial registration number from ClinicalTrials.gov or an equivalent agency.*
Study protocol	*Note where the full trial protocol can be accessed OR if not available, explain why.*
Data collection	*Describe the settings and locales of data collection, noting the time periods of recruitment and data collection.*
Outcomes	*Describe how you pre-defined primary and secondary outcome measures and how you assessed these measures.*
